# Genome-Wide Identification and Expression of the Mulberry PLA Family Under Drought and Salinity

**DOI:** 10.3390/biology15120935

**Published:** 2026-06-15

**Authors:** Wanqi Ma, Lijun Bao, Beining Sun, Mingcheng Li, Xiao Li, Xiaoqing Qin, Feng Jiao, Chao Su, Minjuan Zhang

**Affiliations:** 1 The Sericultural and Silk Research Institute, College of Animal Science and Technology, Northwest A&F University, Yangling 712100, China; 15191015666@163.com (W.M.); baolijun@nwafu.edu.cn (L.B.); s18611874168@163.com (B.S.); lmc0562@163.com (M.L.); qinxq3524@163.com (X.Q.); fjiao@nwsuaf.edu.cn (F.J.); suchao503@126.com (C.S.); 2 College of Life Sciences, Northwest A & F University, Yangling 712100, China; 13207857107@163.com

**Keywords:** *Morus notabilis*, phospholipase A, expression pattern, drought, salinity

## Abstract

Mulberry (*Morus* spp.) is valued for sericulture, medicine, and ecological restoration of degraded lands. Phospholipase A (PLA) enzymes hydrolyze membrane lipids and play critical roles in plant growth and stress responses, yet the *PLA* family in mulberry remains uncharacterized. Here, we performed genome-wide identification of *Morus notabilis* PLA genes in order to systematically analyze their phylogenetic relationships and gene structures, and profile their expression across tissues and under drought and salt stress, thereby providing candidate genes for future functional studies on stress tolerance. Fifty non-redundant *PLA* genes were identified and classified into three subfamilies: *pPLA* (22), *PLA2* (nine), and *PLA1* (19). Most predicted PLA proteins are small (100–500 aa) with predicted instability. Gene structures varied from 1 to 21 exons, and subfamily specific conserved domains (patatin/C2, PLATZ, lipase_3) were detected. Promoters contained stress- and hormone-responsive elements. Expression patterns across five tissues revealed distinct preferential patterns: 56% of genes showed highest expression in roots, with one-fifth in leaves. Under stress, 10 and 12 *MnPLA* genes were increased >2-fold (log_2_FC > 1.0) by drought and salt, respectively. Notably, XP_010108435.1 and XP_024022961.1 exhibited leaf-specific high expression and were salt-induced (log_2_FC > 1.0); XP_010090405.1 (leaf-specific low) was drought-induced (log_2_FC > 1.0); and XP_024023462.1 (root-specific high) was induced by both stresses. These results provide a basis for functional studies and genetic improvement of stress tolerance in mulberry.

## Simple Summary

Mulberry trees are important for silk production and traditional medicine, and exhibit strong resilience to poor soils and frequent pruning, making them an effective candidate for ecological restoration of severely degraded landscapes. In this study, a total of 50 non-redundant *Morus notabilis PLA* members were identified and phylogenetically classified into three subfamilies: *pPLA* (22 members), *PLA2* (nine members), and *PLA1* (19 members). qPCR analysis revealed that the tissue expression patterns of *PLA* genes generally exhibited low transcript levels in leaves and high transcript levels in roots for most genes. Importantly, qPCR analysis revealed that 10 and 12 *PLA* genes were significantly up-regulated (≥2-fold increase with *p* < 0.05) by drought and salt stress, respectively, indicating that these genes represent putative stress-responsive members of the mulberry *PLA* family and serve as candidates for functional studies of stress tolerance.

## 1. Introduction

Mulberry (*Morus* spp.) is a deciduous tree species of the Moraceae family with over 5000 years of cultivation history in China, where it underpins the sericulture industry [1]. Beyond serving as the primary fodder for silkworms, mulberry is also used as livestock feed, in traditional medicine for treating conditions such as diabetes and hypertension, and in ecological restoration for windbreaks, sand fixation, and soil conservation on marginal lands [2,3,4,5]. Understanding how mulberry adapts to drought and salinity is of practical value, yet the contribution of the PLA gene family to these stress responses remains unexplored.

Phospholipase A (PLA) enzymes hydrolyze membrane glycerophospholipids, generating free fatty acids and lysophospholipids that act as signaling molecules or precursors for lipid remodeling [6]. In plants, *PLAs* play critical roles in growth, development, and responses to abiotic stresses such as drought, high salinity, and extreme temperatures [7]. Genome-wide analyses have identified *PLA* family members in diverse species. Gene duplication events have led to expanded PLA families in polyploid crops, such as 29 *pPLA* genes in rapeseed (*Brassica napus*) and 93 *TaPLA* genes in wheat (*Triticum aestivum*) [8,9]. In contrast, monocots like rice and sorghum harbor relatively fewer *PLA* members [10,11]. Phylogenetic studies indicate that *PLA* genes formed major clades before the divergence of monocots and dicots, and most duplicated genes have undergone purifying selection, suggesting conserved functions [8,10,11]. Structural conservation is evident across subfamilies: all *PLA1* members possess a conserved GxSxG lipase motif [6,11,12]; pPLA members contain a patatin domain with an esterase box (GTSTG) and an anion-binding motif (DGGGXRG) [6,11]; and sPLA2 members are characterized by a PA2c domain, a Ca^2+^ binding loop, and a catalytic dyad (His/Asp) [13,14,15].

A growing body of evidence suggests that *PLA* genes are involved in plant abiotic stress responses. In rice, multiple *PLA* genes are induced by salt, cold, and drought [10]. In cotton, silencing *GbPLA1-32* increases salt sensitivity, indicating a positive role in salt tolerance [16]. In maize, *ZmPLA-1γ2* is up-regulated in both roots and shoots under salt stress [17]. Similarly, the flax *LuPLA1* gene family responds strongly to low temperature [18], and *PLA* genes in *Thinopyrum elongatum* are implicated in root salt responses [19]. Moreover, *PLA* family members have been linked to haploid induction in maize and wheat [20,21,22,23], as well as to stress tolerance and yield improvement [16,24]. These findings suggest PLAs may act as potential mediators linking membrane lipid metabolism to cellular signaling, though further validation is required.

Despite extensive studies in many species, the *PLA* gene family in *Morus notabilis* remains poorly characterized. To identify candidate members of the mulberry *PLA* gene family that may play distinct roles in development or stress responses, we systematically identified *PLA* family members in the mulberry genome, analyzed their phylogenetic relationships and gene structures, and quantified their expression across tissues as well as under drought and salt stress using qRT-PCR.

## 2. Materials and Methods

### 2.1. Identification and Characterization of Morus notabilis PLA Genes

We employed Arabidopsis, rice, and poplar PLA sequences as queries to perform a BLAST v2.2.31 search on the *Morus notabilis* genome data available in NCBI (Genome assembly ASM41409v2). The genome-wide screening of potential *Morus notabilis* PLA candidates used HMMER software (v3.0) with a cutoff of e-value ≤ 10^−5^, followed by sequence alignment and domain validation. Putative *Morus notabilis* PLA proteins were further validated for conserved domains using three independent databases: NCBI Batch CD Search (https://www.ncbi.nlm.nih.gov/Structure/bwrpsb/bwrpsb.cgi) (accessed on 13 May 2025), Pfam (http://pfam-legacy.xfam.org/) (accessed on 20 April 2025), and SMART (https://smart.embl.de/) (accessed on 20 April 2025). To visualize the distribution of *PLA* genes on chromosomes, we utilized Tbtools v2.476. Subsequent comprehensive physicochemical characterization was performed using ExPASy (https://web.expasy.org/protparam/) (accessed on 20 April 2025) to determine biochemical properties, including amino acid composition, molecular weight (kDa), theoretical isoelectric point (pI), grand average of hydropathicity (GRAVY), and instability index with default parameters. Subcellular localization predictions were generated using the cello website (http://cello.life.nctu.edu.tw/) (accessed on 21 April 2025).

### 2.2. Conserved Domains, Motifs and Gene Structure Analysis

Conserved domains in *Morus notabilis* PLAs were identified using the Conserved Domain Search Service (NCBI) with an e-value parameter of ≤10^−5^. The protein sequence of *Morus notabilis* phospholipase A was uploaded to the MEME website (https://meme-suite.org/meme/) (accessed on 13 May 2025) to predict conserved motif, with the following parameters: a maximal e-value of 1 × 10^−5^ and a range of motif widths from 6 to 50. The exon–intron structures and intron phases of the predicted PLAs were determined using the Gene Structure Display Server 2.04 with default parameters.

### 2.3. Phylogenetic Analysis and Chromosome Location of Morus notabilis PLAs and AtPLA Sequences

The predicted amino acid sequences of *MnPLA* genes and *PLAs* from Arabidopsis were used to construct a phylogenetic tree. The neighbor-joining (NJ) method was used with 1000 bootstrap replicates in the MEGA v11.0 program. The chromosomal localization of *PLAs* was visualized using TBtools v2.476.

### 2.4. Regulatory Network Analysis of Transcription Factors for Morus notabilis PLAs

To identify the upstream regulatory transcription factors of *MnPLA* genes, promoter sequence data were extracted from 2000 bp upstream of the transcription start site of all *PLA* coding genes in the *Morus notabilis* genome (GCF_000414095.1_ASM41409v2). The potential cis-regulatory elements were scanned and functionally annotated by using the PlantCARE database (http://bioinformatics.psb.ugent.be/webtools/plantcare/html/) (accessed on 21 April 2025). The predictive analysis module of the JASPAR transcription factor database (https://jaspar.elixir.no/) (accessed on 26 May 2025) was used to identify transcription factor binding sites in the promoter sequences.

### 2.5. Plant Materials and Stress Treatment

Mulberry flowers (staminate flower and pistillate flower), fruit, leaves, and roots were sourced from the germplasm resources pool at the Sericulture and Silk Research Institute of Northwest A&F University in Yangling, Shaanxi, China. Leaf, bud, root, and female flower tissues were collected from the mulberry cultivar Hongguo No. 2, while male flowers were collected from the cultivar Neo-Ichinose. The experiment was arranged in a completely randomized design with three independent biological replicates per treatment. Each replicate was a single plant in one pot. Pots were randomly positioned in the greenhouse and rotated weekly to minimize positional effects. Forty-day-old mulberry seedlings were transplanted into plastic pots with a diameter of 25 cm and a height of 20 cm, and were allowed to grow for two weeks. Prior to the induction of drought stress, all plants were watered with 600 mL of water every two days. The plants were grown in a greenhouse under a 14 h light/10 h dark (25 °C) cycle with a relative humidity of 40–50%. The 54-day-old seedlings with 6–8 leaves were used for drought treatment. Drought stress was induced by withholding water for 5 days after a fully watered state, while control plants continued to be watered with 600 mL per pot every two days. The visual scoring of drought injury was performed according to the standard method with minor modifications [25]. The drought injury index (DII) was classified into six grades based on the proportion of wilted, yellowed, or desiccated leaves: 0, no visible symptoms; 1, 1/3 leaves wilted; 2, 2/3 leaves wilted; 3, all leaves wilted or leaf margins yellowed/desiccated; 4, 30% leaves severely yellowed and desiccated; and 5, 50% leaves yellowed and desiccated with abscission. When mulberry leaves exhibited a Grade 2 DII, the second and third leaves from the top of each plant were collected, immediately frozen in liquid nitrogen, and stored at −80 °C until use. For the salt stress treatment, the control group was watered normally, while the salt stress treatment group was irrigated with 300 mM NaCl solution per pot. After 2 days, leaf yellowing symptoms appeared. Total RNA was then extracted from the third leaf taken from the top to the bottom of the plant.

### 2.6. RNA Extraction and Quantitative RT-PCR

Total RNAs were prepared from mulberry leaves using the RNA Extraction Kit (TaKaRa, Dalian, China) according to the manufacturer’s protocol. Genomic DNA was eliminated by treating the extracted RNAs with RNase-free DNase I (TaKaRa, Dalian, China). The first cDNA strand was synthesized using the PrimeScript RT Reagent Kit (TaKaRa, Dalian, China). qRT-PCR was performed using the SYBR^®^ Premix Ex Taq™ II (TaKaRa, Dalian, China) on the Bio-Rad CFX96 Real-Time PCR System (Bio-Rad Laboratories Inc., Hercules, CA, USA). The primers used for qRT-PCR analysis are listed in Supplementary Table S1. Tissue expression levels were calculated using the 2^−ΔCt^ method, and stress-induced levels using the 2^−ΔΔCt^ method. All values were log_2_-transformed. Up-regulation was defined as log_2_FC ≥ 1 and down-regulation as log_2_FC ≤ 0.5. Heatmap display values were clipped to [−2, 2] to mitigate extreme outliers. The full expression data are available in Supplementary Table S4.

## 3. Results

### 3.1. Identification and Chemical and Physical Features of Morus notabilis PLAs

After excluding the redundant genes, 50 *PLAs* were screened in the *Morus notabilis* genome (GCA_012066045.3_ASM1206604v2) using protein sequence alignment and conserved domain screening. The basic biochemical and physicochemical properties of *Morus notabilis PLAs* were analyzed and are listed in Table 1. Based on the bioinformatics results, most PLAs (excluding XP_024024700.1 and XP_024031300.1) were relatively small plant proteins with lengths between 100 and 500 amino acids (aa), which may facilitate rapid synthesis and transport. Most MnPLAs exhibited isoelectric points (pIs) above the average pI of plant proteins (5.62). Stability predictions indicated that 56% of PLAs were unstable, with instability indices above 40, which may be related to their need for rapid degradation or functional regulation in specific physiological processes. Hydrophobicity analysis revealed that only two PLAs (XP_010112575.1 and XP_024020016.1) had positive grand average of hydropathicity (GRAVY) values, suggesting inherent hydrophobicity. Subcellular localization predictions further indicated that PLAs are distributed across multiple compartments, including mitochondria, cytoplasm, chloroplasts, and nuclear, highlighting their potential functional diversity (Table 1). The above results indicate that the *MnPLA* family exhibits significant diversity in physicochemical properties and provide important clues for further exploring the specific mechanisms of *PLAs* in plant life activities.

### 3.2. Gene Structural Features and Conserved Domain of Morus notabilis PLAs

**Figure 1:**
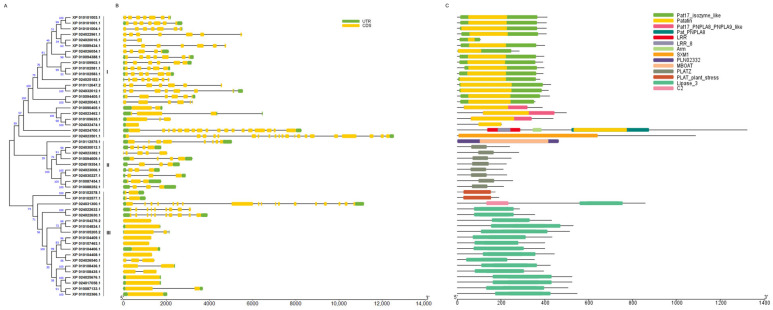
Phylogenetic relationship, gene structure and conserved domain architecture of 50 *MnPLA* genes. (**A**) Neighbor-joining (NJ) phylogenetic tree based on full-length protein sequences. Bootstrap support values (1000 replicates) are shown at nodes in blue font. (**B**) Gene structures: yellow boxes represent exons, black lines represent introns, and green bars indicate 5′/3′ untranslated regions (UTRs). (**C**) Conserved domains: color-coded blocks indicate domain types identified by Pfam. Gene names are listed on the left. This figure illustrates the evolutionary relationships and structural conservation of *MnPLA* genes.

According to the topology of the phylogenetic tree, the *Morus notabilis* phospholipase A gene family predominantly splits into three sub-gene families: *pPLA* (subfamily I), *PLA2* (subfamily II), and *PLA1* (subfamily III). Specifically, 22 *pPLAs* are grouped into subfamily I, nine *PLA2* into subfamily II, and 19 *PLA1* into subfamily III. In terms of evolutionary relationships, *Morus notabilis pPLA* and *PLA2* are closer, while they have a more distant evolutionary relationship with *PLA1* (Figure 1A).

The gene structure of *MnPLA* family members is highly diverse, with the number of exons ranging from 1 to 21 and the number of introns ranging from 0 to 20 (Figure 1B). Significant differences were observed in the number of exons and introns among *PLA* members across the three groups, and also within members of the same group. In Group I, 16 members have exons ranging from five to eight, with one member having only one exon, two members containing two exons, and two members containing three exons. The exception is that XP_024024700.1 and XP_024023501.1 contain 18 and 21 exons, respectively. In Group II, five members had three exons, three members had four exons, and one member had eight exons. In Group III, nine members have only one exon, six members have two exons, one member has three exons, and three members have nine, ten and eighteen exons, respectively. Overall, the diversity in gene structures suggests that *Morus notabilis PLA* family members may have diverse functions in regulating mulberry growth and development.

The full-length amino acid sequences of 50 PLA members were used for conserved domain identification. A total of 14 domains were detected across the PLAs (Figure 1C). The vast majority of PLAs contain one domain, with the exception of XP_024024700.1 and XP_024031300.1, which contain three (Pat_PNPLA8, LRR and Arm) and two domains (lipase_3 and C2), and are annotated as PLA-I and PLA1-PLIP1, respectively. *PLA* members within the same subgroup generally exhibited similar domain compositions. The members of the pPLA subgroup mainly include patatin, Pat17_isozymetlike, Pat17-PNPLA8-PNPLA9_like and Pat-PNPLA8 domains, which belong to the Patatin_and_cPLA2 superfamily (cl11396). cPLA2 may interact with membrane phospholipids or proteins through the C2 domain and may participate in signal transduction by releasing arachidonic acid or similar molecules. Patatin may form complexes with other lipase or regulatory proteins, and is mainly involved in the storage of lipid degradation (such as in tubers) or stress response.

Nine *PLA2* members contain only one PLATZ domain. The PLATZ proteins (plant AT-rich sequence and zinc-binding proteins) are named after their binding to AT-rich DNA sequences. Plant proteins containing PLATZ domains are plant-specific zinc finger protein transcription factors that primarily function to regulate cell proliferation, differentiation, and respond to abiotic stress.

The composition of the *PLA1* subgroup in mulberry trees is relatively simple, except for XP_024031300, which also contains a C2 domain, while the other members only contain a lipase_3 domain. The lipase_3 domain is a typical catalytic domain for triacylglycerol lipase/esterase, and its active center typically contains a catalytic triad composed of serine, aspartic acid/glutamic acid, and histidine. Some proteins containing the lipase_3 domain, such as DAD1/DGL, are key enzymes in jasmonic acid biosynthesis. They release linolenic acid by hydrolyzing membrane lipids, initiating jasmonic acid synthesis and regulating plant defense responses, growth and development, and aging.

There are also two *PLA1* members containing a PLAT_plant_stress domain, and are annotated as LCAT3. PLAT_plant_stress is a subclass of PLAT domains unique to plants which has specialized sequences and specific functions for plant stress response. LCAT3 belongs to the LPCAT family and has a conserved GXSXG catalytic motif and LCAT domain. The promoter region contains multiple stress-response elements. It catalyzes the reacylation of lysophosphatidylcholine, maintains membrane homeostasis and fluidity, responds to stress such as low temperature, drought, and salinity, and enhances plant stress resistance by increasing membrane lipid unsaturation (core function). LCAT3 is an important hub in the plant lipid metabolism network, playing an irreplaceable role in adapting to environmental changes and maintaining normal physiological functions in plants by precisely regulating the fatty acid composition of membrane lipids.

Conservative domains define the core folding mode and potential hydrolytic enzyme activity of proteins. However, proteins containing the same structural domain may exhibit significant differences in their specific functions, such as substrate specificity, subcellular localization, and regulatory mechanisms, across different families.

### 3.3. Phylogenetic Analysis and Chromosome Location of Morus notabilis PLAs

**Figure 2:**
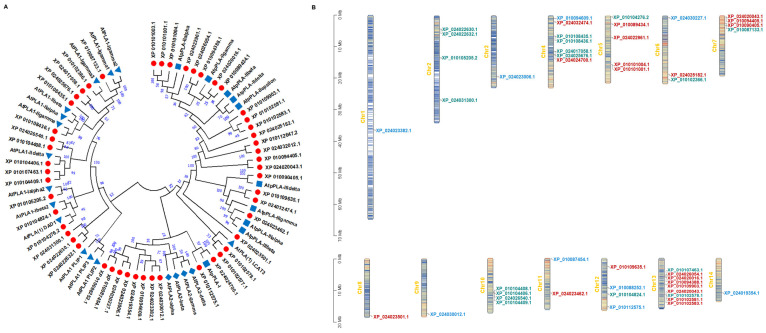
Comparative phylogeny and chromosomal distribution of *MnPLA* genes. (**A**) Phylogenetic tree combining 50 MnPLAs (red circles) and 29 Arabidopsis thaliana PLAs (blue squares for pPLA, blue triangles for PLA1, blue diamonds for PLA2). Orthologous groups are highlighted in different colors. (**B**) Chromosomal localization of *MnPLA* genes on mulberry chromosomes (scales in Mb). Genes are color-coded by subfamily: red for pPLA, blue for PLA2, green for PLA1.

The subgroup classification of PLAs is primarily based on subfamily specific conserved domains, which reflect functional divergence, and secondarily supported by phylogenetic tree topology and homology to Arabidopsis. According to the topology of the phylogenetic tree (Figure 2A), *MnPLAs* and *AtPLAs* (29 *AtPLAs* in total) are clustered into distinct subgroups, reflecting evolutionary divergence and functional classification. The tree reveals clear separation of mulberry *PLAs* and *AtPLAs*, which were grouped into three distinct subclades. Among the three subclades, the *pPLA* subclades contained the largest number of *MnPLA* members, with 22 *pPLAs*, followed by the PLA1 subgroup with 19 *MnPLA1* members, and the *PLA2* subgroup with nine *MnPLA2* members. Within these clades, some mulberry *PLAs* cluster closely with certain Arabidopsis isoforms, suggesting conserved functions or common ancestral origins. However, some Arabidopsis and *Morus notabilis* sequences are often grouped separately, indicating species-specific diversification. Overall, the phylogeny supports the classification of phospholipase genes into well-defined subfamilies and provides insights into the evolutionary relationships between Arabidopsis and *Morus notabilis* phospholipases.

Based on the annotated information (Figure 2B), the chromosome locations of these *MnPLAs* were determined. All of the *MnPLAs* were unevenly distributed across the 14 chromosomes, ranging from one gene on chromosomes 1, 3, 8, 9, and 14 to nine genes on chromosome 13. Additionally, we found that these *PLA* genes are prone to locate on a chromosome region with a relatively high gene density. Moreover, some *MnPLA* genes shared similar chromosome locations and clustered into closed phylogenetic branches.

### 3.4. Conserved Motifs of Morus notabilis PLAs

**Figure 3:**
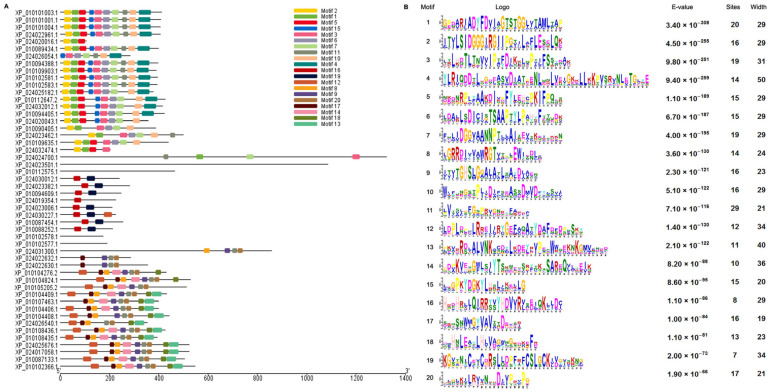
Conserved motif analysis of MnPLAs. (**A**) Distribution of 20 conserved motifs (numbered 1–20, each represented by a colored block) across 50 MnPLAs, as identified by MEME v5.5.1. The length of each block is proportional to the motif length. (**B**) Sequence logos (weblogos) of the 20 motifs, with e-values indicating statistical significance. Key features: Conservation level: Letter height reflects conservation (taller = higher conservation). Amino acid preference: Letter size corresponds to frequency at each position. Chemical property grouping: Color-coded residues (red: acidic; blue: basic; black: hydrophobic, etc.). Information content (bits): Quantifies each position’s contribution to motif specificity.

In total, 20 conserved motifs (motif 1–motif 20) were identified across these *MnPLAs* (Figure 3A), except XP_024023501.1, XP_010112575.1, XP_010102578.1 and XP_010102577.1, which may be because excessive sequence variation did not obtain motif prediction results. As expected, most closely related genes shared similar motif types within each subgroup. Most *pPLAs* have nine motifs; among them, motif 1, motif 3 and motif 7 were most frequently observed, followed by motif 2, motif 10, and motif 6, and then by motif11 and motif 15. Motif 1 contains the serine active site GxSxG, and motif 2 contains the patatin-like PLA2 catalytic DGGGxG (Figure 3B). Motif 3 and motif 6 contain a calcium ion binding ring that forms a C2 domain with DxxDxxD, while motif 7 contains an IDDG sequence located within the catalytic domain of the enzyme and is a core component of the active site, helping to recognize and bind phospholipid molecules and maintain the correct conformation of the catalytic domain. The alignment of *pPLAs* showed that the feature sites (GTSTGG, DGGGI/VRG, DGGVAANNP) contained in motif 1, motif 2, and motif 7 of *pPLAs* are quite conserved.

*MnPLA2* members only have motif 16 and motif 19, and both motif 16 and motif 19 contain phospholipid-binding motifs. Compared with the four members of Arabidopsis *PLA2*, *MnPLA2* predicted two motifs, but was not conservative compared to the motifs of Arabidopsis *PLA2*.

Motifs 9, 11, and 20 emerged as the predominant motifs across all *MnPLA1* members, followed by motif 17, and then motifs 8, 12, 14, and 13. The alignment of *Mn*PLA1s showed that the feature sites (GRRD/ExxxxxRGT and GHSL/MG) contained in motif 8 and motif 9 of *MnPLA1* are quite conserved.

### 3.5. Deciphering Cis-Regulatory Elements and Transcription Factors Upstream of Morus notabilis PLAs

**Figure 4:**
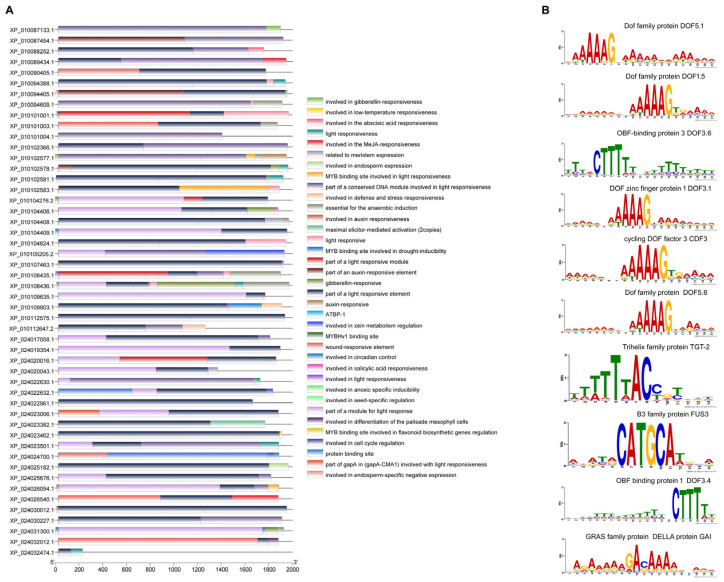
Cis-acting element analysis and transcription factor binding prediction in *MnPLA* promoters. (**A**) Predicted cis-regulatory elements in the 2000 bp upstream promoter regions of *MnPLAs*. Elements are grouped by function (such as stress-responsive, hormone-responsive, light-responsive). (**B**) Top 10 predicted transcription factors with the highest binding probability to *MnPLA* promoters.

By analyzing the promoter regions (2 kb upstream of the start codon) of the 50 *MnPLA* genes, a total of 1430 cis-regulatory elements were identified, representing 36 distinct types (Supplementary Table S2). The promoter regions of *MnPLAs* are predicted to contain abundant stress-induced and hormone-responsive cis-elements. Among these, light-responsive elements were the most prevalent, followed by elements associated with abscisic acid (ABA), methyl jasmonate (MeJA), and gibberellin (GA) responsiveness. Specifically, 16 pPLA, six PLA2, and 12 PLA1 promoters contained only light-responsive elements (Figure 4A). Notably, the promoters also harbor elements responsive to ABA, salicylic acid (SA), auxin, GA, and MeJA—hormones known to mediate plant abiotic stress responses. The presence of these cis-elements suggests a potential involvement of *MnPLA* genes in developmental and stress-related processes, particularly under abiotic stresses such as drought, cold, and salinity. However, these are bioinformatic predictions and require experimental validation (e.g., by promoter–reporter assays or electrophoretic mobility shift assays).

To identify candidate transcription factors (TFs) that may regulate *MnPLA* expression, the promoter sequences were submitted to the JASPAR database, using Arabidopsis as a reference, with a threshold of 99% to select drought-related TFs. The top five TFs with the highest scores of each PLA are listed in Supplementary Table S3. The results predict that drought-related TFs upstream of *MnPLA* genes mainly belong to the Dof2, ATHB-16, and Dof3 families. These predicted TF-binding sites provide testable hypotheses for future validation using methods such as yeast one-hybrid assays, EMSA, or transient expression analysis.

### 3.6. Tissue Specificity and Abiotic Stress-Induced Expression Patterns of Morus notabilis PLAs

**Figure 5:**
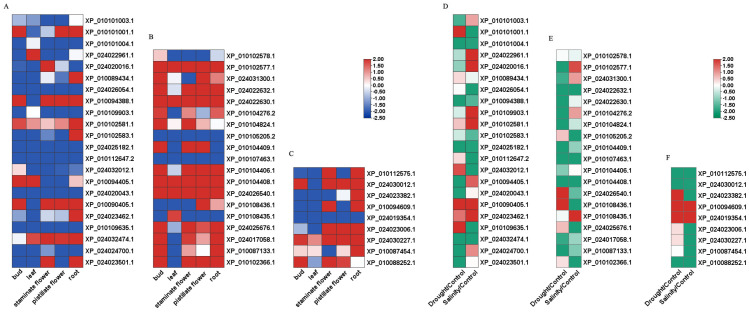
Tissue-specific expression and abiotic stress-responsive expression profiles of *MnPLA* genes. (**A**–**C**) Heatmaps showing log_2_-transformed relative expression levels (2^−ΔCt^ method, normalized to ACTIN) of 50 *MnPLA* genes across five tissues: bud, leaf, staminate flower, pistillate flower, and root. Subfamilies: (**A**) pPLA, (**B**) PLA1, (**C**) PLA2. Color scale: red = high expression, blue = low expression. (**D**–**F**) Heatmaps showing log_2_(fold change) under drought (D/C) and salt (S/C) treatments compared to control (2^−ΔΔCt^ method, normalized to ACTIN). Subfamilies: (**D**) pPLA, (**E**) PLA1, (**F**) PLA2. Color scale: red = induced (log_2_FC ≥ 1, i.e., ≥2−fold up-regulation), green = repressed (log_2_FC ≤ 0.5, i.e., ≤1.5−fold down-regulation), white = no significant change. For visualization, values were clipped to the range [−2, 2] to avoid outlier effects. The full expression data are provided in Supplementary Table S4.

To identify PLA family members potentially involved in tissue-specific functions and stress responses, we quantified the expression levels of 50 *PLA* genes across various tissues (buds, leaves, roots, pistillate flower and staminate flower) using qRT-PCR and visualized them as a heatmap. Across the 50 *MnPLA* genes, distinct tissue-preferential profiles were observed (Figure 5A–C). A majority (28/50, 56%) showed the highest expression in roots, while only 10/50 (20%) peaked in leaves, suggesting a general enrichment of *PLA* transcripts in below-ground organs.

Within the *pPLA* subfamily (Figure 5A), XP_024022961.1 and XP_010094405.1 exhibited leaf-specific high expression (≥4-fold above the root). Conversely, XP_024020016.1 was strongly up-regulated in staminate flowers (8-fold above leaf), and XP_024023462.1 together with XP_010102583.1 showed root-specific expression (≥5-fold above leaf). Notably, three pPLA members (XP_010094388.1, XP_010090405.1, XP_010032474.1) were consistently highly expressed in both staminate flowers and pistillate flowers (reproductive organs), suggesting a potential role in flower development or gametophyte protection.

*PLA1* members (Figure 5B) were generally low in leaves, except for XP_010108435.1. Three *PLA1* genes (XP_010102578.1, XP_010105205.2 and XP_010107463.1) maintained uniformly low expression across all tissues, implying either constitutive housekeeping roles or functional redundancy. XP_010108436.1 exhibited an 8.92-fold higher expression in pistillate flowers compared to the other four tissues, where it was expressed at significantly lower levels.

PLA2 members (Figure 5C) followed a similar root-enriched trend. XP_024030012.1 exhibited marked leaf-specific repression (0.2-fold of root level), while XP_024019354.1 was uniformly low. XP_010087454.1 and XP_024023382.1 were selectively up-regulated in root.

To further explore the functional relevance of these tissue-specific expression patterns, we next examined the responses of *MnPLA* genes to drought and salt stress. Under drought, 10 *MnPLA* genes were induced (log_2_FC ≥ 1.0, *p* < 0.05), and under salt, 12 genes met the same criterion (Figure 5D–F). XP_010108436.1 (pistillate flower-specific) was increased over 3-fold by drought and XP_024020016.1 (staminate flower-specific) was increased over 5-fold by salt, suggesting a role in reproductive organ protection under stress. XP_010090405.1 (leaf-specific) is a strong candidate for further functional validation in leaf drought adaptation. XP_024022961.1 (leaf-specific, up to 6-fold increase) and XP_010108435.1 (leaf-specific, 1.8-fold increase) were significantly increased under salt stress. Notably, XP_024023462.1 (root-specific, about 5-fold induction) was significantly induced by both drought and salt stress, suggesting a convergent stress-responsive role.

In summary, the mulberry *PLA* family contains members with both tissue-preferential and stress-induced expression patterns, pointing to a functional differentiation that likely underlies organ-specific and stress-dependent membrane lipid remodeling. The candidate genes identified above (e.g., XP_024022961.1, XP_010090405.1, XP_024023462.1) provide a prioritized list for future functional studies.

## 4. Discussion

In this study, we identified 50 *PLA* family members in *Morus notabilis* and classified them into three subfamilies (*pPLA*, *PLA2*, and *PLA1*) based on phylogenetic analysis. This classification is consistent with that reported in other plant species, including wheat [9], ginseng [24], rapeseed [8], and flax [18], indicating that the structural framework of the *PLA* family is relatively conserved across angiosperms. However, the exon–intron structures of *MnPLA* genes exhibited remarkable diversity (1–21 exons), with extensive variation even within the same subfamily. For example, among *pPLA* members, the number of exons ranged from 2 to 17, whereas Arabidopsis *pPLAs* show much narrower ranges [26]. Such structural plasticity may underlie functional divergence, representing a unique feature of the mulberry *PLA* family. Notably, the *pPLA* subfamily is the largest in *Morus notabilis* (22 members), a trend also observed in terrestrial plants such as cotton [16], maize [17], and wheat [9]. Nevertheless, mulberry contains nine *PLA2* members, whereas Arabidopsis has only four [27] and rice has six [10], suggesting lineage-specific expansion or retention of *PLA2* genes in mulberry. This expansion may be related to mulberry’s perennial growth habits and repeated pruning–regeneration cycles, which demand active membrane remodeling.

Our qPCR expression profile revealed distinct tissue-preferential patterns. For instance, XP_024022961.1 is highly expressed in leaves, and multiple members (XP_010089434.1, XP_010102583.1, XP_024023462.1, XP_010087454.1) are expressed in root. These patterns are reminiscent of observations in other species: sorghum *pPLA* members are pollen specific [9], maize *PLA1* is predominantly expressed during reproductive growth [11], and *PnPLA1-8* in *Panax notoginseng* is highly expressed in fibrous roots and leaves [24]. Such tissue-restricted expression implies that different *PLA* isoforms fulfill specialized roles in lipid metabolism and signal transduction tailored to the physiological needs of each organ. A unique finding in mulberry is the co-occurrence of root-enriched expression in more than 50% of *MnPLA* genes, which may reflect the species adaptation to rocky, water-limited soils where efficient root membrane remodeling is advantageous.

A major finding of this study is that several tissue-specific *MnPLA* genes show differential expression under drought and salt stress. Specifically, leaf-specific genes (XP_024022961.1 and XP_010090405.1) were induced by salt or drought, the root-specific gene XP_024023462.1 responded to both stresses, and flower-specific members (XP_024020016.1 in staminate flowers under salt; XP_010108436.1 in pistillate flowers under drought) showed distinct stress-responsive patterns. These observations align with previous reports that *PLA* genes are widely responsive to abiotic stresses, as shown in studies of rice *PLA* genes, which are differentially expressed under salt, cold, and drought [10]; silencing *GbPLA1-32* in cotton increases salt sensitivity [16]; flax *LuPLA1* responds strongly to temperature extremes [18]; and maize *pPLA* is up-regulated by salt [17]. The dual specificity (stress + tissue) of *MnPLA* genes allows plants to deploy distinct members in different organs to cope with particular environmental challenges. The candidate genes identified here provide a prioritized list for functional validation.

Beyond stress responses, PLA enzymes are deeply involved in development. Since the first report of PLA1 activity in maple vacuolar membranes [28], numerous studies have established that *pPLA* members regulate cell elongation. Overexpression of *AtpPLAIIIβ* and *AtpPLAIIIδ* in Arabidopsis causes dwarfism and shortened cells, linked to altered cellulose or lignin content [26,29,30,31]. Similarly, rice *OsPLAIIIα* controls longitudinal growth of vegetative organs and seeds [32], and this function is conserved in poplar and ginseng [33,34,35]. Given that *MnpPLA* members share high sequence similarity with their Arabidopsis homologs, they may perform analogous roles, a possibility that deserves experimental validation.

Regarding lipid metabolism, *pPLAIIIδ* increases seed oil content and promotes long-chain fatty acid accumulation in Arabidopsis and *Brassica campestris* [30,36]. *RcpPLAIIIβ* from castor bean participates in removing hydroxy fatty acids from phosphatidylcholine [36]. In rice, expression differences in *OsPLA* genes correlate with grain oil content [10]. In ginseng, overexpression of *PgpPLAIIIβ* unexpectedly reduced ginsenosides while increasing beneficial fatty acids [35]. The predominance of *pPLA* members in *Morus notabilis* suggests potential applications in improving oil quality, though direct evidence is lacking.

For male reproduction, the Arabidopsis pollen-specific *sPLA2* is essential for pollen development [31]. *Morus notabilis* contains eight *PLA2* members, six of which are highly expressed in male flowers, implying conserved roles in male reproductive development. More strikingly, mutations in pollen-specific *pPLA* genes (NLD/MTL) in maize induce maternal haploidy [37,38,39,40], a breakthrough for crop breeding. The rice homolog *OsMATL* also triggers haploid induction when mutated [38]. In *Morus notabilis*, four *pPLA* genes (XP_010094388.1, XP_024025182.1, XP_010112647.2, XP_010090405.1) are highly expressed in male flowers and share homology with MATL. Whether these genes are functionally involved in male reproductive development or haploid induction remains an open and urgent question.

To validate the roles of candidate *MnPLA* genes, heterologous overexpression in Arabidopsis or tobacco may be used to test for altered stress tolerance, growth, or reproductive phenotypes; biochemical assays (recombinant protein expression for PLA enzyme activit and lipidomics to identify substrate specificity) may be completed; and promoter–reporter fusions may be feasible to confirm tissue-specific and stress-inducible cis-elements.

## 5. Conclusions

This study provides the first systematic characterization of the *Morus notabilis PLA* family, revealing unique structural diversity, subfamily expansion patterns, and dual tissue-/stress-specific expression profiles. This work establishes a basis for future functional dissection and genetic improvement of stress tolerance in this economically important tree species, though experimental validation through protein abundance analysis, enzymatic activity assays, and transgenic approaches such as overexpression in model plants will be required.

## Figures and Tables

**Table 1:** Physicochemical properties and predicted subcellular localization of 50 *PLA* genes identified from the *Morus notabilis* genome.

	Protein Name	Gene ID	Accession Number	Size (aa)	Molecular Weight (kDa)	pI	Instability Index	Aliphatic Index	GRAVY	Subcellular Localization
1	pPLA-IIalpha	21396365	XP_010101003.1	411	45,176.6	6.1	34.98	95.69	−0.145	Cytoplasm
2	pPLA-IIalpha	21396363	XP_010101001.1	408	44,688.86	6.03	34.93	93.5	−0.208	Cytoplasm
3	pPLA-IIalpha	21396366	XP_010101004.1	407	44,292.52	6.22	29.23	92.29	−0.174	Cytoplasm
4	pPLA-IIalpha	21408747	XP_024022961.1	407	44,946.18	5.19	33.78	95.87	−0.204	Cytoplasm
5	pPLA-IIgamma	112091214	XP_024020016.1	106	11,601.44	5.23	36.44	108.58	0.016	Chloroplast
6	pPLA-IIgamma	21386294	XP_010089434.1	399	44,469.4	5.72	34.51	87.97	−0.363	Cytoplasm
7	pPLA-IIgamma	21395000	XP_024026054.1	284	31,019.02	8.65	21.53	86.94	−0.29	Mitochondria
8	pPLA-IIgamma	21391647	XP_010094388.1	397	43,206.18	6.61	28.21	92.9	−0.159	Plasma Membrane
9	pPLA-IIbeta	21396421	XP_010109903.1	390	43,029.71	5.8	28.84	86.85	−0.317	Mitochondria
10	pPLA-IIdelta	21387470	XP_010102581.1	396	43,278.32	6.05	27	96.36	−0.203	Cytoplasm
11	pPLA-IIepsilon	21387472	XP_010102583.1	393	43,057.14	7.67	27.9	84.99	−0.196	Cytoplasm
12	pPLA-II	21407268	XP_024025182.1	378	41,828.04	4.93	36.54	99.05	−0.116	Cytoplasm
13	pPLA-II	21402053	XP_010112647.2	427	48,169.97	5	42.09	84.07	−0.295	Cytoplasm
14	pPLA-II	21402052	XP_024032012.1	416	46,665.11	4.94	35.28	82.07	−0.327	Cytoplasm
15	pPLA-II	21401736	XP_010094405.1	422	47,174.75	7.14	38.18	81.18	−0.379	Cytoplasm
16	pPLA-II	21401737	XP_024020043.1	357	38,888.42	6.03	28.25	89.94	−0.199	Cytoplasm
17	pPLA-IIIdelta	21407788	XP_010090405.1	389	42,182.2	8.06	34.68	88.46	−0.173	Chloroplast
18	pPLA-IIIgamma	21396583	XP_024023462.1	499	54,443.66	5.92	40.21	75.49	−0.267	Chloroplast
19	pPLA-IIIdelta	21398678	XP_010109635.1	439	47,184.83	8.98	43.71	86.04	−0.197	Mitochondria
20	pPLA-IIIdelta	112095021	XP_024032474.1	203	21,760.98	9.49	45.37	84.24	−0.084	Chloroplast
21	PLA-I	21396066	XP_024024700.1	1323	145,799.89	5.76	49.44	94.75	−0.124	Nuclear
22	pPLA-III	21407839	XP_024023501.1	1087	121,075.52	4.66	50.68	100.63	−0.036	Plasma Membrane
23	PLA-I	21393870	XP_010112575.1	464	52,365.52	9.34	42.52	104.63	0.321	Plasma Membrane
24	PLA2-delta	21403746	XP_024030012.1	240	26,940.14	8.79	65.38	86.88	−0.163	Extracellular
25	PLA2-gamma	112092183	XP_024023382.1	282	31,918.51	8.71	54.15	71.21	−0.57	Nuclear
26	PLA2-beta	21393169	XP_010094609.1	247	27,475.38	7.02	42.91	81.3	−0.251	Nuclear
27	PLA2-alpha	21390182	XP_024019354.1	225	25,788.93	9.15	41.93	75.82	−0.483	Nuclear
28	PLA2-delta	21405427	XP_024023006.1	210	23,730.15	9.05	69.98	75.19	−0.463	Nuclear
29	PLA2-gamma	21395001	XP_024030227.1	226	25,524.22	8.81	63.89	78.54	−0.472	Nuclear
30	PLA2-beta	21402592	XP_010087454.1	254	28,772.65	8.61	64.28	59.88	−0.689	Nuclear
31	PLA2-alpha	21386756	XP_010088252.1	213	24,183.7	9.29	62.23	67.7	−0.564	Nuclear
32	PLA1-LCAT3	21387467	XP_010102578.1	174	19,491.81	5.09	42.13	81.72	−0.274	Extracellular
33	PLA1-LCAT3	21387466	XP_010102577.1	190	20,898.62	5.48	36.73	87.63	−0.164	Extracellular
34	PLA1-PLIP1	21394616	XP_024031300.1	857	95,690.24	5.4	42.9	84.28	−0.356	Cytoplasm
35	PLA1-PLIP2	21406333	XP_024022632.1	285	32,622.32	6.55	40.3	89.58	−0.063	Plasma Membrane
36	PLA1-PLIP3	21406333	XP_024022630.1	355	40,400.15	6.4	37.19	87.55	−0.111	Plasma Membrane
37	DAD1	21388847	XP_010104276.2	430	48,394.7	8.96	46.41	86.35	−0.43	Mitochondria
38	PLA1-Ibeta2	21406774	XP_010104824.1	529	59,140.65	8.55	50.89	83.31	−0.421	Mitochondria
39	PLA1-Ialpha2	21394313	XP_010105205.2	512	57,463.37	6.33	41.78	86.23	−0.188	Plasma Membrane
40	PLA1-IIdelta	21393656	XP_010104409.1	432	48,550.99	5.62	30	82.59	−0.326	Cytoplasm
41	PLA1-IIdelta	21399254	XP_010107463.1	399	45,208.99	8.81	44.55	75.24	−0.583	Mitochondria
42	PLA1-IIdelta	21393653	XP_010104406.1	400	45,126.04	5.38	44	85.27	−0.293	Cytoplasm
43	PLA1-IIdelta	21393655	XP_010104408.1	443	50,409.28	5.95	44.82	88.83	−0.381	Cytoplasm
44	PLA1-IIdelta	21393654	XP_024026540.1	353	40,058.5	5.64	32.56	86.37	−0.381	Cytoplasm
45	PLA1-IIgamma	21389599	XP_010108436.1	426	48,306.75	8.74	35.64	81.95	−0.524	Mitochondria Cytoplasm
46	PLA1-II	21389598	XP_010108435.1	394	44,467.31	5.41	22.82	86.4	−0.387	Cytoplasm
47	PLA1-Igamma	112092822	XP_024025676.1	524	59,519.4	8.11	46.12	79.83	−0.439	Mitochondria
48	PLA1-Igamma	112090306	XP_024017058.1	524	59,390.24	8.11	46.25	79.83	−0.438	Mitochondria
49	PLA1-Igamma	21395771	XP_010087133.1	505	57,626.54	7.31	49.23	83.6	−0.399	Mitochondria
50	PLA1-Igamma3	21398418	XP_010102366.1	547	62,132.28	7.34	45.27	76.97	−0.552	Cytoplasm

Note: aa, amino acid; pI, isoelectric point; GRAVY, grand average of hydropathy. Physicochemical parameters were computed using the ExPASy ProtParam tool. (https://web.expasy.org/protparam/) (accessed on 20 April 2025). Subcellular localization was predicted by cello (http://cello.life.nctu.edu.tw/) (accessed on 21 April 2025). Accession numbers correspond to transcript/protein sequences in the *Morus notabilis* genome database (GenBank GCA_000414095.2_ASM41409v2).

## Data Availability

The original contributions presented in this study are included in the article/Supplementary Material. Further inquiries can be directed to the corresponding author.

## Supplementary Materials

The following supporting information can be downloaded at: https://www.mdpi.com/article/10.3390/biology15120935/s1, Table S1: Primer sequences used for quantitative real-time PCR (qRT-PCR) analysis in this study; Table S2: Positions and functional categories of cis-acting elements in the promoter regions of 50 *Morus notabilis PLA* genes; Table S3: Predicted transcription factors, matched sequences, binding sites in *Morus notabilis PLA* promoter regions; Table S4: Log_2_-transformed expression values of 50 *Morus notabilis PLA* genes across five tissues (bud, leaf, staminate flower, pistillate flower, root) and under drought and salt stress treatments, grouped by subfamily (*pPLA*, *PLA2*, *PLA1*). Tissue expression values are 2^−ΔCt^ (log_2_-transformed); stress values are 2^−ΔΔCt^ (log_2_-transformed). Data are organized by subfamily: *pPLA* (22 genes), *PLA2* (9 genes), *PLA1* (19 genes). These values were used to generate the heatmaps in Figure 5.
