# Supplementary material for: Genome-Wide Identification and Expression of the Mulberry PLA Family Under Drought and Salinity
Source: Biology (Basel). 2026 Jun 15;15(12):935. doi: 10.3390/biology15120935 (PMC13295505; doi:10.3390/biology15120935)
Supplement: Supplementary file 1 [file biology-15-00935-s001.zip › Supplementary Table S1-S4.pdf]

Supplementary Table S1. Primer sequences used for quantitative real-time PCR (qRT-PCR) analysis in this study.

Number	Gene name	Primer sequences (5' to 3')	Primer sequences (3' to 5')
1	pPLA-IIalpha	ATTGATGGCGGTGTT	ATCGGCGTTGTATTT
2	pPLA-IIalpha	TGGTGGCATAAGAGG	TCGTTTGGAGCAGTAA
3	pPLA-IIalpha	AAGCAACAACACCCAA	AGCAACGCCACCA
4	pPLA-IIalpha	TATTCCAAGCCCTCC	CAACGGTTTCTTCAGTA
5	pPLA-IIgamma	AGATGGGAAAGATGTGA	TTGCTTGTGGGAAAA
6	pPLA-IIgamma	CACCAGACGCCAACA	CAACAACCGAAGCA
7	pPLA-IIgamma	CAGCCTCAGCATTAGA	AAGACGGATTATTAGCA
8	pPLA-IIgamma	TGAGGCTTGCTGATT	AAACCCACTTGCTTG
9	pPLA-IIbeta	TGGAGGTGGCATTAG	TCCTTGGCAGCGTA
10	pPLA-IIdelta	CTCCCACTTATCTCCC	GTTTATCGCCAGCAT
11	pPLA-IIepsilon	GATAAGGACGGAAAGAC	CAAACACCCAACTGAA
12	pPLA-II	CCAAGAGGGACGAA	CCAGAGGACCTAAAGA
13	pPLA-II	GGGTTTGTCTTGATTT	TTCGGACCAGTTGC
14	pPLA-II	AATAATCCGACTGTAGC	TTTCCACCCTTTCC
15	pPLA-II	AATGGTCGTCCACTTC	TATGCTACTCGGCTCT
16	pPLA-II	TGGCTGCCGAGAA	TGGTGCCTGGGATA
17	pPLA-IIIdelta	CACTGCGAAACCACCA	CCCTTGCGTTGAATAGA
18	pPLA-IIIgamma	GCGTGACATACAAGGAAA	CGTAGCCGAACAGGAA

19	pPLA-IIIdelta	GTTCCCGCTCTGTAATG	TTCCCAGCCACCTTG
20	pPLA-IIIdelta	GGCATCGCCGACTACTT	TCCTCAGCGGTGAACAT
21	PLA-I	CGAGTTACGGGTTCT	GCCAGCAAAGGGT
22	pPLA-III	TTCTAACCGACACGC	ACATTTCGCCTCGT
23	PLA-I	CCATTCCCGTGAGC	CGTAACCTAGCAGCAT
24	PLA2-delta	ATGTCATAAGAACGGAAGG	CGGTAAGCCGAAACG
25	PLA2-gamma	GGGTCTGGCAACTAT	GTCGTCTCCCATTTC
26	PLA2-beta	TTGGTGGTTGTGGG	TGGAGAAGAGGGTGAG
27	PLA2-alpha	GCTCATTGCCTTCAT	GGCGGGTCTTGTAT
28	PLA2-delta	CTTCTATTGCCGTTCAT	CTCCCACAGATTTCACA
29	PLA2-gamma	TAGAACTGCCGTCAAA	CTTCCCTACTTCCCAC
30	PLA2-beta	AACACCTGCCTCGTC	TTGGCTCGCATTTT
31	PLA2-alpha	GCGTCACCAACACCT	GCCGCCATTTGCTTC
32	PLA1-LCAT3	CCGACGATGTGGAGAAGT	TGGCAGCGGATGTG
33	PLA1-LCAT3	TCTCATCGGACGGCTAC	AACTGCTGCTGGTGGC
34	PLA1-PLIP1	ATACCGACAGTCCCA	ATAACCATCTCCCAAA
35	PLA1-PLIP2	TTGTGCCTCATTTGC	CTACAGGTTGGGTCTT
36	PLA1-PLIP3	CTCACAGCGTTGGTTT	TGCTACAGGTTGGGTC
37	DAD1	AGCAACAACTGGGAGG	AGAGGTGGCGTAGAGG
38	PLA1-Ibeta2	ATCGGTTCTACTCTTCCTG	GCGGGTCTATTGCTTT

39	PLA1-Ialpha2	CGCCTGCTACAAGAC	GATGACACCGCCACA
40	PLA1-IIdelta	AGCAACAACTGGGAGG	AGAGGTGGCGTAGAGG
41	PLA1-IIdelta	CGGTGACCCGACAGAT	CGCCAGGCAACATAGAC
42	PLA1-IIdelta	GGCTTACTATTTACACTTCG	CTACTTGCGGACACCC
43	PLA1-IIdelta	CACAATGCCATCCAGG	GCTCAGAGTCGCCAAA
44	PLA1-IIdelta	GCCTCCGACTACCAAG	AAATCCGTAATCCACTCC
45	PLA1-IIgamma	TCAGCGTTCTCGTTTC	GTTTGGGCTTGTTGTAT
46	PLA1-II	GCGAAAGGAACGAA	TAACGCCGCATAAA
47	PLA1-Igamma	ATAGGGCAAAGGGTG	TCTGAAGGCGGTGTT
48	PLA1-Igamma	ATAGGGCAAAGGGTG	TCTGAAGGCGGTGTT
49	PLA1-Igamma	CGGCGAGGGAACAGAT	CCGAGGACCCGAAAA
50	PLA1-Igamma3	CACCACAACCACCATCA	ACGAGTCCCTCCCAGTC

Supplementary Table S2. Positions and functional categories of cis-acting elements in the promoter regions of 50 *Morus notabilis PLA* genes.

Gene name	start	stop	Element type
XP_010087133.1	1901	27	involved in gibberellin-responsiveness
XP_010087133.1	1567	26	involved in low-temperature responsiveness
XP_010087133.1	981	25	involved in the abscisic acid responsiveness
XP_010087133.1	1451	25	involved in the abscisic acid responsiveness
XP_010087133.1	1451	26	light responsiveness

XP_010087133.1	980	26	light responsiveness
XP_010087133.1	67	25	involved in the MeJA-responsiveness
XP_010087133.1	1313	25	involved in the MeJA-responsiveness
XP_010087133.1	67	25	involved in the MeJA-responsiveness
XP_010087133.1	1313	25	involved in the MeJA-responsiveness
XP_010087133.1	1298	26	related to meristem expression
XP_010087133.1	239	27	involved in endosperm expression
XP_010087133.1	1553	28.5	involved in differentiation of the palisade mesophyll cells
XP_010087133.1	-4	27	gibberellin-responsive
XP_010087133.1	1628	27	MYB binding site involved in light responsiveness
XP_010087133.1	1151	26	part of a conserved DNA module involved in light responsiveness
XP_010087133.1	1780	26	part of a conserved DNA module involved in light responsiveness
XP_010087454.1	904	29	involved in defense and stress responsiveness
XP_010087454.1	1006	26	involved in low-temperature responsiveness
XP_010087454.1	739	25	involved in the abscisic acid responsiveness
XP_010087454.1	-16	26	essential for the anaerobic induction
XP_010087454.1	156	26	essential for the anaerobic induction
XP_010087454.1	1271	27	involved in auxin responsiveness
XP_010087454.1	738	26	light responsiveness
XP_010087454.1	736	28	light responsiveness
XP_010087454.1	43	25	involved in the MeJA-responsiveness
XP_010087454.1	1092	25	involved in the MeJA-responsiveness
XP_010087454.1	43	25	involved in the MeJA-responsiveness
XP_010087454.1	1092	25	involved in the MeJA-responsiveness
XP_010087454.1	794	29	maximal elicitor-mediated activation (2copies)
XP_010087454.1	1814	29	maximal elicitor-mediated activation (2copies)

XP_010087454.1	1407	26	light responsive
XP_010087454.1	985	26	MYB binding site involved in drought-inducibility
XP_010087454.1	1924	26	part of a conserved DNA module involved in light responsiveness
XP_010087454.1	390	33	part of a light responsive module
XP_010087454.1	1092	28	part of an auxin-responsive element
XP_010088252.1	1712	29	involved in defense and stress responsiveness
XP_010088252.1	468	25	involved in the abscisic acid responsiveness
XP_010088252.1	1468	26	involved in the abscisic acid responsiveness
XP_010088252.1	1469	25	involved in the abscisic acid responsiveness
XP_010088252.1	1760	29	involved in the abscisic acid responsiveness
XP_010088252.1	619	26	essential for the anaerobic induction
XP_010088252.1	1014	26	essential for the anaerobic induction
XP_010088252.1	467	26	light responsiveness
XP_010088252.1	1468	26	light responsiveness
XP_010088252.1	1468	26	light responsiveness
XP_010088252.1	55	25	involved in the MeJA-responsiveness
XP_010088252.1	586	25	involved in the MeJA-responsiveness
XP_010088252.1	55	25	involved in the MeJA-responsiveness
XP_010088252.1	586	25	involved in the MeJA-responsiveness
XP_010088252.1	63	27	involved in endosperm expression
XP_010088252.1	985	27	gibberellin-responsive
XP_010088252.1	1404	27	light responsive
XP_010088252.1	1405	26	light responsive
XP_010088252.1	538	26	light responsive
XP_010088252.1	664	26	light responsive
XP_010088252.1	178	26	part of a conserved DNA module involved in light responsiveness

XP_010088252.1	241	26	part of a conserved DNA module involved in light responsiveness
XP_010088252.1	910	26	part of a conserved DNA module involved in light responsiveness
XP_010088252.1	1129	26	part of a conserved DNA module involved in light responsiveness
XP_010088252.1	1578	26	part of a conserved DNA module involved in light responsiveness
XP_010088252.1	1627	26	part of a conserved DNA module involved in light responsiveness
XP_010088252.1	567	27	part of a light responsive element
XP_010088252.1	1163	26	part of a light responsive element
XP_010089434.1	586	26	auxin-responsive
XP_010089434.1	1428	25	involved in the abscisic acid responsiveness
XP_010089434.1	1721	25	involved in the abscisic acid responsiveness
XP_010089434.1	1431	26	essential for the anaerobic induction
XP_010089434.1	1471	26	essential for the anaerobic induction
XP_010089434.1	1536	26	essential for the anaerobic induction
XP_010089434.1	1544	26	essential for the anaerobic induction
XP_010089434.1	451	27	involved in auxin responsiveness
XP_010089434.1	1427	26	light responsiveness
XP_010089434.1	1721	26	light responsiveness
XP_010089434.1	427	25	involved in the MeJA-responsiveness
XP_010089434.1	591	25	involved in the MeJA-responsiveness
XP_010089434.1	843	25	involved in the MeJA-responsiveness
XP_010089434.1	1723	25	involved in the MeJA-responsiveness
XP_010089434.1	1948	25	involved in the MeJA-responsiveness
XP_010089434.1	427	25	involved in the MeJA-responsiveness
XP_010089434.1	591	25	involved in the MeJA-responsiveness
XP_010089434.1	843	25	involved in the MeJA-responsiveness
XP_010089434.1	1723	25	involved in the MeJA-responsiveness

XP_010089434.1	1948	25	involved in the MeJA-responsiveness
XP_010089434.1	1141	26	related to meristem expression
XP_010089434.1	835	27	involved in endosperm expression
XP_010089434.1	1208	27	involved in endosperm expression
XP_010089434.1	629	28.5	involved in differentiation of the palisade mesophyll cells
XP_010089434.1	1165	27	gibberellin-responsive
XP_010089434.1	866	27	light responsive
XP_010089434.1	867	26	light responsive
XP_010089434.1	168	26	part of a conserved DNA module involved in light responsiveness
XP_010089434.1	356	26	part of a conserved DNA module involved in light responsiveness
XP_010089434.1	435	26	part of a conserved DNA module involved in light responsiveness
XP_010089434.1	1755	26	part of a conserved DNA module involved in light responsiveness
XP_010089434.1	557	29	part of a light responsive element
XP_010090405.1	1282	26	auxin-responsive
XP_010090405.1	324	30	ATBP-1
XP_010090405.1	977	29	involved in defense and stress responsiveness
XP_010090405.1	1137	29	involved in defense and stress responsiveness
XP_010090405.1	1315	29	involved in defense and stress responsiveness
XP_010090405.1	1070	26	involved in low-temperature responsiveness
XP_010090405.1	1729	25	involved in the abscisic acid responsiveness
XP_010090405.1	254	26	essential for the anaerobic induction
XP_010090405.1	1246	26	essential for the anaerobic induction
XP_010090405.1	1260	26	essential for the anaerobic induction
XP_010090405.1	1729	26	light responsiveness
XP_010090405.1	1022	25	involved in the MeJA-responsiveness
XP_010090405.1	1300	25	involved in the MeJA-responsiveness

XP_010090405.1	1022	25	involved in the MeJA-responsiveness
XP_010090405.1	1300	25	involved in the MeJA-responsiveness
XP_010090405.1	418	29	involved in zein metabolism regulation
XP_010090405.1	1498	29	involved in zein metabolism regulation
XP_010090405.1	1241	26	related to meristem expression
XP_010090405.1	165	26	light responsive
XP_010090405.1	551	26	light responsive
XP_010090405.1	1761	26	light responsive
XP_010090405.1	858	26	MYB binding site involved in drought-inducibility
XP_010090405.1	1523	26	MYBHv1 binding site
XP_010090405.1	958	29	part of a conserved DNA module involved in light responsiveness
XP_010090405.1	129	26	part of a conserved DNA module involved in light responsiveness
XP_010090405.1	323	26	part of a conserved DNA module involved in light responsiveness
XP_010090405.1	479	26	part of a conserved DNA module involved in light responsiveness
XP_010090405.1	605	26	part of a conserved DNA module involved in light responsiveness
XP_010090405.1	748	26	part of a conserved DNA module involved in light responsiveness
XP_010090405.1	1432	28	part of a light responsive element
XP_010090405.1	1690	28	part of a light responsive element
XP_010090405.1	941	29	part of a light responsive element
XP_010090405.1	1438	30	part of a light responsive element
XP_010090405.1	1769	30	part of a light responsive element
XP_010090405.1	942	29	part of a light responsive element
XP_010090405.1	977	26	part of a light responsive element
XP_010090405.1	1350	26	part of a light responsive element
XP_010090405.1	1436	26	part of a light responsive element
XP_010090405.1	1776	26	part of a light responsive element

XP_010090405.1	710	29	wound-responsive element
XP_010094388.1	1383	26	auxin-responsive
XP_010094388.1	1428	26	auxin-responsive
XP_010094388.1	1754	29	involved in defense and stress responsiveness
XP_010094388.1	503	25	involved in the abscisic acid responsiveness
XP_010094388.1	613	25	involved in the abscisic acid responsiveness
XP_010094388.1	1942	25	involved in the abscisic acid responsiveness
XP_010094388.1	57	26	essential for the anaerobic induction
XP_010094388.1	966	26	essential for the anaerobic induction
XP_010094388.1	1089	26	essential for the anaerobic induction
XP_010094388.1	433	26	light responsiveness
XP_010094388.1	460	26	light responsiveness
XP_010094388.1	500	28	light responsiveness
XP_010094388.1	552	26	light responsiveness
XP_010094388.1	557	26	light responsiveness
XP_010094388.1	502	26	light responsiveness
XP_010094388.1	613	26	light responsiveness
XP_010094388.1	1941	26	light responsiveness
XP_010094388.1	113	25	involved in the MeJA-responsiveness
XP_010094388.1	590	25	involved in the MeJA-responsiveness
XP_010094388.1	113	25	involved in the MeJA-responsiveness
XP_010094388.1	590	25	involved in the MeJA-responsiveness
XP_010094388.1	1440	28	involved in zein metabolism regulation
XP_010094388.1	990	27	involved in endosperm expression
XP_010094388.1	1729	27	involved in endosperm expression
XP_010094388.1	1313	26	light responsive

XP_010094388.1	1839	26	light responsive
XP_010094388.1	637	26	MYB binding site involved in drought-inducibility
XP_010094388.1	1115	27	MYB binding site involved in light responsiveness
XP_010094388.1	495	26	part of a conserved DNA module involved in light responsiveness
XP_010094388.1	681	26	part of a conserved DNA module involved in light responsiveness
XP_010094388.1	1392	26	part of a conserved DNA module involved in light responsiveness
XP_010094388.1	1533	26	part of a conserved DNA module involved in light responsiveness
XP_010094388.1	1157	30	part of a light responsive element
XP_010094388.1	1783	29	part of a light responsive element
XP_010094388.1	1103	28	part of a light responsive element
XP_010094388.1	1157	30	part of a light responsive element
XP_010094388.1	1158	28	part of a light responsive element
XP_010094405.1	59	26	auxin-responsive
XP_010094405.1	1292	26	auxin-responsive
XP_010094405.1	1225	29	involved in defense and stress responsiveness
XP_010094405.1	1743	29	involved in defense and stress responsiveness
XP_010094405.1	1957	27	involved in gibberellin-responsiveness
XP_010094405.1	1125	25	involved in the abscisic acid responsiveness
XP_010094405.1	1792	30	involved in circadian control
XP_010094405.1	1123	29	light responsiveness
XP_010094405.1	1124	26	light responsiveness
XP_010094405.1	1317	26	light responsiveness
XP_010094405.1	1344	26	light responsiveness
XP_010094405.1	1434	26	light responsiveness
XP_010094405.1	92	25	involved in the MeJA-responsiveness
XP_010094405.1	92	25	involved in the MeJA-responsiveness

XP_010094405.1	134	29	involved in zein metabolism regulation
XP_010094405.1	75	27	involved in endosperm expression
XP_010094405.1	1561	27	gibberellin-responsive
XP_010094405.1	1635	27	gibberellin-responsive
XP_010094405.1	1948	30	light responsive
XP_010094405.1	4	26	light responsive
XP_010094405.1	262	26	part of a conserved DNA module involved in light responsiveness
XP_010094405.1	1194	26	part of a conserved DNA module involved in light responsiveness
XP_010094405.1	1649	26	part of a conserved DNA module involved in light responsiveness
XP_010094405.1	581	29.5	part of a light responsive element
XP_010094405.1	1941	29	part of a light responsive element
XP_010094405.1	1657	26	part of a light responsive element
XP_010094405.1	1670	26	part of a light responsive element
XP_010094405.1	1086	31	part of an auxin-responsive element
XP_010094609.1	728	26	involved in low-temperature responsiveness
XP_010094609.1	1044	29	involved in salicylic acid responsiveness
XP_010094609.1	1246	25	involved in the abscisic acid responsiveness
XP_010094609.1	594	26	essential for the anaerobic induction
XP_010094609.1	997	26	essential for the anaerobic induction
XP_010094609.1	1914	26	essential for the anaerobic induction
XP_010094609.1	81	26	light responsiveness
XP_010094609.1	1245	26	light responsiveness
XP_010094609.1	1134	29	involved in zein metabolism regulation
XP_010094609.1	1670	27	light responsive
XP_010094609.1	1671	26	light responsive
XP_010094609.1	46	26	part of a conserved DNA module involved in light responsiveness

XP_010094609.1	1452	26	part of a conserved DNA module involved in light responsiveness
XP_010094609.1	1650	26	part of a conserved DNA module involved in light responsiveness
XP_010101001.1	1541	30	involved in defense and stress responsiveness
XP_010101001.1	384	25	involved in the abscisic acid responsiveness
XP_010101001.1	1336	25	involved in the abscisic acid responsiveness
XP_010101001.1	1448	26	involved in the abscisic acid responsiveness
XP_010101001.1	1449	25	involved in the abscisic acid responsiveness
XP_010101001.1	1784	25	involved in the abscisic acid responsiveness
XP_010101001.1	196	26	essential for the anaerobic induction
XP_010101001.1	209	26	essential for the anaerobic induction
XP_010101001.1	395	26	essential for the anaerobic induction
XP_010101001.1	111	29	involved in circadian control
XP_010101001.1	1591	29	involved in circadian control
XP_010101001.1	1448	26	light responsiveness
XP_010101001.1	13	26	light responsiveness
XP_010101001.1	45	26	light responsiveness
XP_010101001.1	382	28	light responsiveness
XP_010101001.1	383	26	light responsiveness
XP_010101001.1	599	26	light responsiveness
XP_010101001.1	1336	26	light responsiveness
XP_010101001.1	1448	26	light responsiveness
XP_010101001.1	1508	26	light responsiveness
XP_010101001.1	1783	26	light responsiveness
XP_010101001.1	40	25	involved in the MeJA-responsiveness
XP_010101001.1	74	25	involved in the MeJA-responsiveness
XP_010101001.1	189	25	involved in the MeJA-responsiveness

XP_010101001.1	40	25	involved in the MeJA-responsiveness
XP_010101001.1	74	25	involved in the MeJA-responsiveness
XP_010101001.1	189	25	involved in the MeJA-responsiveness
XP_010101001.1	1000	29	involved in zein metabolism regulation
XP_010101001.1	501	27	gibberellin-responsive
XP_010101001.1	520	27	gibberellin-responsive
XP_010101001.1	1313	27	gibberellin-responsive
XP_010101001.1	1896	27	gibberellin-responsive
XP_010101001.1	1968	27	light responsive
XP_010101001.1	1969	26	light responsive
XP_010101001.1	229	26	MYBHv1 binding site
XP_010101001.1	296	26	part of a conserved DNA module involved in light responsiveness
XP_010101001.1	924	28	part of a light responsive element
XP_010101001.1	1129	29	part of a light responsive element
XP_010101001.1	1422	26	part of a light responsive element
XP_010101001.1	424	33	part of a light responsive module
XP_010101001.1	1135	33	part of a light responsive module
XP_010101003.1	1751	30	ATBP-1
XP_010101003.1	1874	30	ATBP-1
XP_010101003.1	1288	27	involved in gibberellin-responsiveness
XP_010101003.1	1638	26	involved in low-temperature responsiveness
XP_010101003.1	589	25	involved in the abscisic acid responsiveness
XP_010101003.1	1875	26	essential for the anaerobic induction
XP_010101003.1	589	26	light responsiveness
XP_010101003.1	1700	25	involved in the MeJA-responsiveness
XP_010101003.1	1700	25	involved in the MeJA-responsiveness

XP_010101003.1	1608	26	related to meristem expression
XP_010101003.1	1038	26	light responsive
XP_010101003.1	1046	26	MYBHv1 binding site
XP_010101003.1	106	26	part of a conserved DNA module involved in light responsiveness
XP_010101003.1	124	26	part of a conserved DNA module involved in light responsiveness
XP_010101003.1	1420	26	part of a conserved DNA module involved in light responsiveness
XP_010101003.1	1538	26	part of a conserved DNA module involved in light responsiveness
XP_010101003.1	1553	26	part of a conserved DNA module involved in light responsiveness
XP_010101003.1	1712	26	part of a conserved DNA module involved in light responsiveness
XP_010101003.1	1732	28	part of a light responsive element
XP_010101003.1	437	29	wound-responsive element
XP_010101003.1	868	29	wound-responsive element
XP_010101004.1	1101	29	involved in light responsiveness
XP_010101004.1	923	26	involved in low-temperature responsiveness
XP_010101004.1	815	25	involved in the abscisic acid responsiveness
XP_010101004.1	1042	26	essential for the anaerobic induction
XP_010101004.1	814	26	light responsiveness
XP_010101004.1	829	26	involved in anoxic specific inducibility
XP_010101004.1	826	26	light responsive
XP_010101004.1	1244	27	MYB binding site involved in light responsiveness
XP_010101004.1	1316	26	MYBHv1 binding site
XP_010101004.1	146	28	part of a conserved DNA module involved in light responsiveness
XP_010101004.1	315	26	part of a conserved DNA module involved in light responsiveness
XP_010101004.1	376	26	part of a conserved DNA module involved in light responsiveness
XP_010101004.1	1408	26	part of a conserved DNA module involved in light responsiveness
XP_010102366.1	269	26	auxin-responsive

XP_010102366.1	105	29	involved in defense and stress responsiveness
XP_010102366.1	1242	29	involved in defense and stress responsiveness
XP_010102366.1	809	29	involved in salicylic acid responsiveness
XP_010102366.1	687	26	involved in the abscisic acid responsiveness
XP_010102366.1	688	25	involved in the abscisic acid responsiveness
XP_010102366.1	350	26	essential for the anaerobic induction
XP_010102366.1	844	26	essential for the anaerobic induction
XP_010102366.1	1169	26	essential for the anaerobic induction
XP_010102366.1	684	29	light responsiveness
XP_010102366.1	687	26	light responsiveness
XP_010102366.1	687	26	light responsiveness
XP_010102366.1	838	25	involved in the MeJA-responsiveness
XP_010102366.1	1761	25	involved in the MeJA-responsiveness
XP_010102366.1	838	25	involved in the MeJA-responsiveness
XP_010102366.1	1761	25	involved in the MeJA-responsiveness
XP_010102366.1	1800	27	involved in endosperm expression
XP_010102366.1	913	28.5	involved in differentiation of the palisade mesophyll cells
XP_010102366.1	1699	27	light responsive
XP_010102366.1	1700	26	light responsive
XP_010102366.1	343	26	MYB binding site involved in drought-inducibility
XP_010102366.1	1954	26	MYB binding site involved in drought-inducibility
XP_010102366.1	358	26	part of a conserved DNA module involved in light responsiveness
XP_010102366.1	362	26	part of a conserved DNA module involved in light responsiveness
XP_010102366.1	601	26	part of a conserved DNA module involved in light responsiveness
XP_010102366.1	1850	26	part of a conserved DNA module involved in light responsiveness
XP_010102366.1	1875	26	part of a conserved DNA module involved in light responsiveness

XP_010102366.1	1961	26	part of a conserved DNA module involved in light responsiveness
XP_010102366.1	744	29	part of a light responsive element
XP_010102577.1	899	26	auxin-responsive
XP_010102577.1	1950	26	auxin-responsive
XP_010102577.1	0	27	involved in gibberellin-responsiveness
XP_010102577.1	1750	26	essential for the anaerobic induction
XP_010102577.1	1036	27	involved in endosperm expression
XP_010102577.1	1362	27	involved in endosperm expression
XP_010102577.1	676	29	maximal elicitor-mediated activation (2copies)
XP_010102577.1	1065	29	maximal elicitor-mediated activation (2copies)
XP_010102577.1	1689	27	MYB binding site involved in light responsiveness
XP_010102577.1	1610	29	part of a conserved DNA module involved in light responsiveness
XP_010102577.1	130	26	part of a conserved DNA module involved in light responsiveness
XP_010102577.1	1226	26	part of a conserved DNA module involved in light responsiveness
XP_010102578.1	624	26	auxin-responsive
XP_010102578.1	800	30	ATBP-1
XP_010102578.1	1670	26	involved in low-temperature responsiveness
XP_010102578.1	1475	26	involved in the abscisic acid responsiveness
XP_010102578.1	1476	25	involved in the abscisic acid responsiveness
XP_010102578.1	1965	25	involved in the abscisic acid responsiveness
XP_010102578.1	525	26	essential for the anaerobic induction
XP_010102578.1	574	26	essential for the anaerobic induction
XP_010102578.1	1475	26	light responsiveness
XP_010102578.1	389	26	light responsiveness
XP_010102578.1	1475	26	light responsiveness
XP_010102578.1	1964	26	light responsiveness

XP_010102578.1	138	25	involved in the MeJA-responsiveness
XP_010102578.1	138	25	involved in the MeJA-responsiveness
XP_010102578.1	217	26	related to meristem expression
XP_010102578.1	1603	29	maximal elicitor-mediated activation (2copies)
XP_010102578.1	1501	27	gibberellin-responsive
XP_010102578.1	1891	27	gibberellin-responsive
XP_010102578.1	1186	26	part of a conserved DNA module involved in light responsiveness
XP_010102578.1	226	30	part of a light responsive element
XP_010102578.1	1818	27	part of a light responsive element
XP_010102578.1	633	26	part of a light responsive element
XP_010102578.1	1146	26	part of a light responsive element
XP_010102578.1	1196	26	part of a light responsive element
XP_010102578.1	135	28	part of an auxin-responsive element
XP_010102581.1	734	26	auxin-responsive
XP_010102581.1	527	29	involved in defense and stress responsiveness
XP_010102581.1	362	27	involved in gibberellin-responsiveness
XP_010102581.1	782	29	involved in salicylic acid responsiveness
XP_010102581.1	958	29	involved in salicylic acid responsiveness
XP_010102581.1	268	25	involved in the abscisic acid responsiveness
XP_010102581.1	319	25	involved in the abscisic acid responsiveness
XP_010102581.1	719	27	involved in the abscisic acid responsiveness
XP_010102581.1	1924	25	involved in the abscisic acid responsiveness
XP_010102581.1	27	26	essential for the anaerobic induction
XP_010102581.1	444	26	essential for the anaerobic induction
XP_010102581.1	822	26	essential for the anaerobic induction
XP_010102581.1	1439	26	essential for the anaerobic induction

XP_010102581.1	1482	26	essential for the anaerobic induction
XP_010102581.1	416	27	involved in auxin responsiveness
XP_010102581.1	265	29	light responsiveness
XP_010102581.1	268	26	light responsiveness
XP_010102581.1	1923	26	light responsiveness
XP_010102581.1	318	26	light responsiveness
XP_010102581.1	493	28	involved in seed-specific regulation
XP_010102581.1	737	29	involved in zein metabolism regulation
XP_010102581.1	1801	27	gibberellin-responsive
XP_010102581.1	747	26	MYB binding site involved in drought-inducibility
XP_010102581.1	1025	26	part of a conserved DNA module involved in light responsiveness
XP_010102581.1	1059	26	part of a conserved DNA module involved in light responsiveness
XP_010102581.1	1243	28	part of a light responsive element
XP_010102581.1	1781	28	part of a light responsive element
XP_010102581.1	7	30	part of a light responsive element
XP_010102581.1	871	26	part of a light responsive element
XP_010102583.1	1312	26	auxin-responsive
XP_010102583.1	1370	26	auxin-responsive
XP_010102583.1	1695	29	involved in defense and stress responsiveness
XP_010102583.1	635	26	involved in low-temperature responsiveness
XP_010102583.1	1427	26	involved in low-temperature responsiveness
XP_010102583.1	1462	26	involved in low-temperature responsiveness
XP_010102583.1	1588	26	involved in low-temperature responsiveness
XP_010102583.1	1884	29	involved in salicylic acid responsiveness
XP_010102583.1	1152	25	involved in the abscisic acid responsiveness
XP_010102583.1	305	26	essential for the anaerobic induction

XP_010102583.1	787	26	essential for the anaerobic induction
XP_010102583.1	1152	26	light responsiveness
XP_010102583.1	162	25	involved in the MeJA-responsiveness
XP_010102583.1	693	25	involved in the MeJA-responsiveness
XP_010102583.1	162	25	involved in the MeJA-responsiveness
XP_010102583.1	693	25	involved in the MeJA-responsiveness
XP_010102583.1	1459	26	involved in anoxic specific inducibility
XP_010102583.1	309	27	gibberellin-responsive
XP_010102583.1	618	27	gibberellin-responsive
XP_010102583.1	474	26	light responsive
XP_010102583.1	547	26	light responsive
XP_010102583.1	1892	26	light responsive
XP_010102583.1	1377	26	MYB binding site involved in drought-inducibility
XP_010102583.1	1801	27	MYB binding site involved in light responsiveness
XP_010102583.1	1047	30	part of a light responsive element
XP_010104276.2	859	31	ATBP-1
XP_010104276.2	1783	29	involved in defense and stress responsiveness
XP_010104276.2	262	26	involved in low-temperature responsiveness
XP_010104276.2	515	26	involved in low-temperature responsiveness
XP_010104276.2	226	25	involved in the abscisic acid responsiveness
XP_010104276.2	406	25	involved in the abscisic acid responsiveness
XP_010104276.2	427	25	involved in the abscisic acid responsiveness
XP_010104276.2	450	25	involved in the abscisic acid responsiveness
XP_010104276.2	598	25	involved in the abscisic acid responsiveness
XP_010104276.2	971	25	involved in the abscisic acid responsiveness
XP_010104276.2	1136	25	involved in the abscisic acid responsiveness

XP_010104276.2	1319	27	involved in the abscisic acid responsiveness
XP_010104276.2	1472	25	involved in the abscisic acid responsiveness
XP_010104276.2	280	26	essential for the anaerobic induction
XP_010104276.2	818	29	involved in circadian control
XP_010104276.2	867	29	involved in circadian control
XP_010104276.2	426	26	light responsiveness
XP_010104276.2	598	26	light responsiveness
XP_010104276.2	971	26	light responsiveness
XP_010104276.2	995	30	light responsiveness
XP_010104276.2	1135	26	light responsiveness
XP_010104276.2	1471	26	light responsiveness
XP_010104276.2	225	26	light responsiveness
XP_010104276.2	406	26	light responsiveness
XP_010104276.2	449	26	light responsiveness
XP_010104276.2	39	28	involved in seed-specific regulation
XP_010104276.2	408	25	involved in the MeJA-responsiveness
XP_010104276.2	448	25	involved in the MeJA-responsiveness
XP_010104276.2	1457	25	involved in the MeJA-responsiveness
XP_010104276.2	1782	25	involved in the MeJA-responsiveness
XP_010104276.2	408	25	involved in the MeJA-responsiveness
XP_010104276.2	448	25	involved in the MeJA-responsiveness
XP_010104276.2	1457	25	involved in the MeJA-responsiveness
XP_010104276.2	1782	25	involved in the MeJA-responsiveness
XP_010104276.2	953	29	involved in zein metabolism regulation
XP_010104276.2	937	26	involved in anoxic specific inducibility
XP_010104276.2	1439	26	involved in anoxic specific inducibility

XP_010104276.2	0	27	gibberellin-responsive
XP_010104276.2	1636	27	gibberellin-responsive
XP_010104276.2	1211	26	light responsive
XP_010104276.2	1753	26	light responsive
XP_010104276.2	1312	29	part of a conserved DNA module involved in light responsiveness
XP_010104276.2	312	26	part of a conserved DNA module involved in light responsiveness
XP_010104276.2	1793	27	part of a light responsive element
XP_010104276.2	1239	33	part of a light responsive module
XP_010104276.2	1085	28	part of a module for light response
XP_010104406.1	1591	30	ATBP-1
XP_010104406.1	1038	26	involved in low-temperature responsiveness
XP_010104406.1	1532	26	involved in low-temperature responsiveness
XP_010104406.1	-9	29	involved in salicylic acid responsiveness
XP_010104406.1	1798	29	involved in salicylic acid responsiveness
XP_010104406.1	1880	29	involved in salicylic acid responsiveness
XP_010104406.1	930	25	involved in the abscisic acid responsiveness
XP_010104406.1	1780	25	involved in the abscisic acid responsiveness
XP_010104406.1	401	26	essential for the anaerobic induction
XP_010104406.1	1488	26	essential for the anaerobic induction
XP_010104406.1	791	27	involved in auxin responsiveness
XP_010104406.1	929	26	light responsiveness
XP_010104406.1	1779	26	light responsiveness
XP_010104406.1	1014	28	involved in seed-specific regulation
XP_010104406.1	1180	25	involved in the MeJA-responsiveness
XP_010104406.1	1432	25	involved in the MeJA-responsiveness
XP_010104406.1	1180	25	involved in the MeJA-responsiveness

XP_010104406.1	1432	25	involved in the MeJA-responsiveness
XP_010104406.1	85	29	involved in zein metabolism regulation
XP_010104406.1	1025	26	related to meristem expression
XP_010104406.1	1112	27	involved in endosperm expression
XP_010104406.1	886	27	gibberellin-responsive
XP_010104406.1	477	27	gibberellin-responsive
XP_010104406.1	1849	27	gibberellin-responsive
XP_010104406.1	520	26	MYB binding site involved in drought-inducibility
XP_010104406.1	1424	26	MYB binding site involved in drought-inducibility
XP_010104406.1	655	26	MYBHv1 binding site
XP_010104406.1	784	26	part of a conserved DNA module involved in light responsiveness
XP_010104406.1	734	28	part of a light responsive element
XP_010104406.1	1620	29	part of a light responsive element
XP_010104406.1	1618	29	part of a light responsive element
XP_010104406.1	-17	26	part of a light responsive element
XP_010104406.1	1082	26	part of a light responsive element
XP_010104406.1	1321	26	part of a light responsive element
XP_010104406.1	523	28	part of a module for light response
XP_010104406.1	1065	28	part of a module for light response
XP_010104408.1	871	26	auxin-responsive
XP_010104408.1	881	26	auxin-responsive
XP_010104408.1	1035	29	involved in defense and stress responsiveness
XP_010104408.1	440	26	involved in low-temperature responsiveness
XP_010104408.1	1967	26	involved in low-temperature responsiveness
XP_010104408.1	885	25	involved in the abscisic acid responsiveness
XP_010104408.1	1677	25	involved in the abscisic acid responsiveness

XP_010104408.1	238	26	essential for the anaerobic induction
XP_010104408.1	1970	26	essential for the anaerobic induction
XP_010104408.1	1676	26	light responsiveness
XP_010104408.1	884	26	light responsiveness
XP_010104408.1	1030	25	involved in the MeJA-responsiveness
XP_010104408.1	1219	25	involved in the MeJA-responsiveness
XP_010104408.1	1510	25	involved in the MeJA-responsiveness
XP_010104408.1	1030	25	involved in the MeJA-responsiveness
XP_010104408.1	1219	25	involved in the MeJA-responsiveness
XP_010104408.1	1510	25	involved in the MeJA-responsiveness
XP_010104408.1	705	29	involved in zein metabolism regulation
XP_010104408.1	1932	26	related to meristem expression
XP_010104408.1	205	26	involved in anoxic specific inducibility
XP_010104408.1	903	26	involved in anoxic specific inducibility
XP_010104408.1	1225	26	involved in anoxic specific inducibility
XP_010104408.1	1535	27	gibberellin-responsive
XP_010104408.1	1650	27	gibberellin-responsive
XP_010104408.1	492	26	light responsive
XP_010104408.1	748	26	MYB binding site involved in drought-inducibility
XP_010104408.1	1020	26	MYBHv1 binding site
XP_010104408.1	357	26	part of a conserved DNA module involved in light responsiveness
XP_010104408.1	618	26	part of a conserved DNA module involved in light responsiveness
XP_010104408.1	104	29	part of a light responsive element
XP_010104408.1	1769	29	part of a light responsive element
XP_010104408.1	1209	30	part of a light responsive element
XP_010104409.1	681	30	ATBP-1

XP_010104409.1	77	26	involved in low-temperature responsiveness
XP_010104409.1	1593	26	involved in low-temperature responsiveness
XP_010104409.1	300	29	involved in salicylic acid responsiveness
XP_010104409.1	726	29	involved in salicylic acid responsiveness
XP_010104409.1	1	25	involved in the abscisic acid responsiveness
XP_010104409.1	132	27	involved in the abscisic acid responsiveness
XP_010104409.1	610	29	involved in the abscisic acid responsiveness
XP_010104409.1	663	26	involved in the abscisic acid responsiveness
XP_010104409.1	664	25	involved in the abscisic acid responsiveness
XP_010104409.1	824	25	involved in the abscisic acid responsiveness
XP_010104409.1	1775	25	involved in the abscisic acid responsiveness
XP_010104409.1	353	26	essential for the anaerobic induction
XP_010104409.1	663	26	light responsiveness
XP_010104409.1	1774	26	light responsiveness
XP_010104409.1	1	26	light responsiveness
XP_010104409.1	663	26	light responsiveness
XP_010104409.1	823	26	light responsiveness
XP_010104409.1	1536	29	light responsiveness
XP_010104409.1	1772	28	light responsiveness
XP_010104409.1	1368	28	involved in seed-specific regulation
XP_010104409.1	290	25	involved in the MeJA-responsiveness
XP_010104409.1	765	25	involved in the MeJA-responsiveness
XP_010104409.1	822	25	involved in the MeJA-responsiveness
XP_010104409.1	290	25	involved in the MeJA-responsiveness
XP_010104409.1	765	25	involved in the MeJA-responsiveness
XP_010104409.1	822	25	involved in the MeJA-responsiveness

XP_010104409.1	203	27	gibberellin-responsive
XP_010104409.1	755	26	MYB binding site involved in drought-inducibility
XP_010104409.1	1639	26	MYB binding site involved in drought-inducibility
XP_010104409.1	1715	26	part of a conserved DNA module involved in light responsiveness
XP_010104409.1	1655	27	part of a light responsive element
XP_010104409.1	856	26	part of a light responsive element
XP_010104409.1	1956	26	part of a light responsive element
XP_010104409.1	1402	28	part of a module for light response
XP_010104824.1	1756	26	auxin-responsive
XP_010104824.1	1081	27	involved in gibberellin-responsiveness
XP_010104824.1	1683	26	involved in low-temperature responsiveness
XP_010104824.1	1173	29	involved in salicylic acid responsiveness
XP_010104824.1	1852	29	involved in salicylic acid responsiveness
XP_010104824.1	432	25	involved in the abscisic acid responsiveness
XP_010104824.1	485	25	involved in the abscisic acid responsiveness
XP_010104824.1	799	25	involved in the abscisic acid responsiveness
XP_010104824.1	1788	25	involved in the abscisic acid responsiveness
XP_010104824.1	1795	25	involved in the abscisic acid responsiveness
XP_010104824.1	675	26	essential for the anaerobic induction
XP_010104824.1	1020	26	essential for the anaerobic induction
XP_010104824.1	432	26	light responsiveness
XP_010104824.1	485	26	light responsiveness
XP_010104824.1	799	26	light responsiveness
XP_010104824.1	1788	26	light responsiveness
XP_010104824.1	1795	26	light responsiveness
XP_010104824.1	417	25	involved in the MeJA-responsiveness

XP_010104824.1	434	25	involved in the MeJA-responsiveness
XP_010104824.1	541	25	involved in the MeJA-responsiveness
XP_010104824.1	801	25	involved in the MeJA-responsiveness
XP_010104824.1	1136	25	involved in the MeJA-responsiveness
XP_010104824.1	1797	25	involved in the MeJA-responsiveness
XP_010104824.1	417	25	involved in the MeJA-responsiveness
XP_010104824.1	434	25	involved in the MeJA-responsiveness
XP_010104824.1	541	25	involved in the MeJA-responsiveness
XP_010104824.1	801	25	involved in the MeJA-responsiveness
XP_010104824.1	1136	25	involved in the MeJA-responsiveness
XP_010104824.1	1797	25	involved in the MeJA-responsiveness
XP_010104824.1	798	29	involved in zein metabolism regulation
XP_010104824.1	1673	27	gibberellin-responsive
XP_010104824.1	853	26	light responsive
XP_010104824.1	1946	27	light responsive
XP_010104824.1	999	26	MYB binding site involved in drought-inducibility
XP_010104824.1	1058	26	MYB binding site involved in drought-inducibility
XP_010104824.1	143	29	part of a conserved DNA module involved in light responsiveness
XP_010104824.1	55	26	part of a conserved DNA module involved in light responsiveness
XP_010104824.1	643	26	part of a conserved DNA module involved in light responsiveness
XP_010104824.1	951	26	part of a conserved DNA module involved in light responsiveness
XP_010104824.1	992	26	part of a conserved DNA module involved in light responsiveness
XP_010104824.1	1604	27	part of a light responsive element
XP_010104824.1	880	28	part of a light responsive element
XP_010105205.2	1936	27	involved in gibberellin-responsiveness
XP_010105205.2	235	26	involved in low-temperature responsiveness

XP_010105205.2	604	26	involved in low-temperature responsiveness
XP_010105205.2	841	25	involved in the abscisic acid responsiveness
XP_010105205.2	1488	26	involved in the abscisic acid responsiveness
XP_010105205.2	1489	25	involved in the abscisic acid responsiveness
XP_010105205.2	1500	26	involved in the abscisic acid responsiveness
XP_010105205.2	1501	25	involved in the abscisic acid responsiveness
XP_010105205.2	600	26	essential for the anaerobic induction
XP_010105205.2	841	26	light responsiveness
XP_010105205.2	1488	26	light responsiveness
XP_010105205.2	1497	29	light responsiveness
XP_010105205.2	1500	26	light responsiveness
XP_010105205.2	1488	26	light responsiveness
XP_010105205.2	1500	26	light responsiveness
XP_010105205.2	797	25	involved in the MeJA-responsiveness
XP_010105205.2	1238	25	involved in the MeJA-responsiveness
XP_010105205.2	1342	25	involved in the MeJA-responsiveness
XP_010105205.2	1584	25	involved in the MeJA-responsiveness
XP_010105205.2	797	25	involved in the MeJA-responsiveness
XP_010105205.2	1238	25	involved in the MeJA-responsiveness
XP_010105205.2	1342	25	involved in the MeJA-responsiveness
XP_010105205.2	1584	25	involved in the MeJA-responsiveness
XP_010105205.2	1929	29	involved in zein metabolism regulation
XP_010105205.2	120	26	light responsive
XP_010105205.2	943	26	MYB binding site involved in drought-inducibility
XP_010105205.2	457	26	part of a conserved DNA module involved in light responsiveness
XP_010105205.2	1266	26	part of a conserved DNA module involved in light responsiveness

XP_010105205.2	420	28	part of a module for light response
XP_010107463.1	237	26	auxin-responsive
XP_010107463.1	1726	26	auxin-responsive
XP_010107463.1	1315	30	ATBP-1
XP_010107463.1	1338	30	ATBP-1
XP_010107463.1	106	29	involved in defense and stress responsiveness
XP_010107463.1	816	29	involved in defense and stress responsiveness
XP_010107463.1	1926	29	involved in light responsiveness
XP_010107463.1	693	26	involved in low-temperature responsiveness
XP_010107463.1	835	29	involved in salicylic acid responsiveness
XP_010107463.1	1376	29	involved in salicylic acid responsiveness
XP_010107463.1	394	25	involved in the abscisic acid responsiveness
XP_010107463.1	461	25	involved in the abscisic acid responsiveness
XP_010107463.1	1706	27	involved in the abscisic acid responsiveness
XP_010107463.1	581	29	involved in circadian control
XP_010107463.1	393	26	light responsiveness
XP_010107463.1	391	28	light responsiveness
XP_010107463.1	461	26	light responsiveness
XP_010107463.1	385	25	involved in the MeJA-responsiveness
XP_010107463.1	463	25	involved in the MeJA-responsiveness
XP_010107463.1	530	25	involved in the MeJA-responsiveness
XP_010107463.1	663	25	involved in the MeJA-responsiveness
XP_010107463.1	666	25	involved in the MeJA-responsiveness
XP_010107463.1	385	25	involved in the MeJA-responsiveness
XP_010107463.1	463	25	involved in the MeJA-responsiveness
XP_010107463.1	530	25	involved in the MeJA-responsiveness

XP_010107463.1	663	25	involved in the MeJA-responsiveness
XP_010107463.1	666	25	involved in the MeJA-responsiveness
XP_010107463.1	539	26	MYBHv1 binding site
XP_010107463.1	899	26	part of a conserved DNA module involved in light responsiveness
XP_010107463.1	1035	28	part of a light responsive element
XP_010107463.1	1372	28	part of a light responsive element
XP_010107463.1	410	27	part of a light responsive element
XP_010107463.1	1164	26	part of a light responsive element
XP_010107463.1	1910	26	part of a light responsive element
XP_010108435.1	859	29	involved in defense and stress responsiveness
XP_010108435.1	2	29	involved in light responsiveness
XP_010108435.1	700	29	involved in salicylic acid responsiveness
XP_010108435.1	484	25	involved in the abscisic acid responsiveness
XP_010108435.1	937	25	involved in the abscisic acid responsiveness
XP_010108435.1	992	26	involved in the abscisic acid responsiveness
XP_010108435.1	993	25	involved in the abscisic acid responsiveness
XP_010108435.1	1293	26	involved in the abscisic acid responsiveness
XP_010108435.1	1294	25	involved in the abscisic acid responsiveness
XP_010108435.1	297	26	essential for the anaerobic induction
XP_010108435.1	321	26	essential for the anaerobic induction
XP_010108435.1	386	26	essential for the anaerobic induction
XP_010108435.1	1902	26	essential for the anaerobic induction
XP_010108435.1	481	29	light responsiveness
XP_010108435.1	484	26	light responsiveness
XP_010108435.1	584	26	light responsiveness
XP_010108435.1	937	26	light responsiveness

XP_010108435.1	992	26	light responsiveness
XP_010108435.1	1291	29	light responsiveness
XP_010108435.1	1293	26	light responsiveness
XP_010108435.1	992	26	light responsiveness
XP_010108435.1	1293	26	light responsiveness
XP_010108435.1	1003	25	involved in the MeJA-responsiveness
XP_010108435.1	1003	25	involved in the MeJA-responsiveness
XP_010108435.1	1226	26	related to meristem expression
XP_010108435.1	1214	29	maximal elicitor-mediated activation (2copies)
XP_010108435.1	12	28.5	involved in differentiation of the palisade mesophyll cells
XP_010108435.1	1367	27	gibberellin-responsive
XP_010108435.1	1322	31	light responsive
XP_010108435.1	1475	26	light responsive
XP_010108435.1	146	27	MYB binding site involved in light responsiveness
XP_010108435.1	1265	26	part of a conserved DNA module involved in light responsiveness
XP_010108435.1	1418	26	part of a conserved DNA module involved in light responsiveness
XP_010108435.1	483	29	part of a light responsive element
XP_010108435.1	1068	30	part of a light responsive element
XP_010108435.1	1196	30	part of a light responsive element
XP_010108435.1	1197	28	part of a light responsive element
XP_010108435.1	859	26	part of a light responsive element
XP_010108435.1	890	26	part of a light responsive element
XP_010108435.1	952	33	part of a light responsive module
XP_010108435.1	362	29.5	protein binding site
XP_010108436.1	1006	26	auxin-responsive
XP_010108436.1	1054	26	auxin-responsive

XP_010108436.1	1162	26	auxin-responsive
XP_010108436.1	179	29	involved in defense and stress responsiveness
XP_010108436.1	355	29	involved in defense and stress responsiveness
XP_010108436.1	711	26	involved in low-temperature responsiveness
XP_010108436.1	1586	25	involved in the abscisic acid responsiveness
XP_010108436.1	25	26	essential for the anaerobic induction
XP_010108436.1	694	26	essential for the anaerobic induction
XP_010108436.1	1410	26	essential for the anaerobic induction
XP_010108436.1	1675	26	essential for the anaerobic induction
XP_010108436.1	1974	26	essential for the anaerobic induction
XP_010108436.1	1038	30	light responsiveness
XP_010108436.1	1041	26	light responsiveness
XP_010108436.1	1141	26	light responsiveness
XP_010108436.1	1585	26	light responsiveness
XP_010108436.1	1027	25	involved in the MeJA-responsiveness
XP_010108436.1	1322	25	involved in the MeJA-responsiveness
XP_010108436.1	1027	25	involved in the MeJA-responsiveness
XP_010108436.1	1322	25	involved in the MeJA-responsiveness
XP_010108436.1	11	27	gibberellin-responsive
XP_010108436.1	1515	27	gibberellin-responsive
XP_010108436.1	787	27	light responsive
XP_010108436.1	857	27	light responsive
XP_010108436.1	82	26	part of a conserved DNA module involved in light responsiveness
XP_010108436.1	192	26	part of a conserved DNA module involved in light responsiveness
XP_010108436.1	221	26	part of a conserved DNA module involved in light responsiveness
XP_010108436.1	768	26	part of a conserved DNA module involved in light responsiveness

XP_010108436.1	801	26	part of a conserved DNA module involved in light responsiveness
XP_010108436.1	782	27	part of a light responsive element
XP_010108436.1	658	26	part of a light responsive element
XP_010108436.1	431	28	part of a module for light response
XP_010109635.1	1365	26	auxin-responsive
XP_010109635.1	321	26	involved in low-temperature responsiveness
XP_010109635.1	1383	26	essential for the anaerobic induction
XP_010109635.1	1453	26	essential for the anaerobic induction
XP_010109635.1	1548	27	involved in auxin responsiveness
XP_010109635.1	205	26	light responsiveness
XP_010109635.1	348	26	light responsiveness
XP_010109635.1	610	26	light responsiveness
XP_010109635.1	615	26	light responsiveness
XP_010109635.1	1052	29	light responsiveness
XP_010109635.1	1338	29	light responsiveness
XP_010109635.1	988	25	involved in the MeJA-responsiveness
XP_010109635.1	988	25	involved in the MeJA-responsiveness
XP_010109635.1	286	29	involved in zein metabolism regulation
XP_010109635.1	315	27	light responsive
XP_010109635.1	655	26	light responsive
XP_010109635.1	735	26	light responsive
XP_010109635.1	1091	26	light responsive
XP_010109635.1	1491	27	light responsive
XP_010109635.1	1492	26	light responsive
XP_010109635.1	737	27	MYB binding site involved in light responsiveness
XP_010109635.1	1093	27	MYB binding site involved in light responsiveness

XP_010109635.1	244	30	part of a light responsive element
XP_010109635.1	1771	28	part of a light responsive element
XP_010109635.1	1754	26	part of a light responsive element
XP_010109635.1	1610	28	part of a module for light response
XP_010109903.1	275	28.5	involved in cell cycle regulation
XP_010109903.1	65	29	involved in defense and stress responsiveness
XP_010109903.1	106	29	involved in defense and stress responsiveness
XP_010109903.1	319	29	involved in defense and stress responsiveness
XP_010109903.1	1911	29	involved in defense and stress responsiveness
XP_010109903.1	664	26	involved in low-temperature responsiveness
XP_010109903.1	101	29	involved in salicylic acid responsiveness
XP_010109903.1	63	26	essential for the anaerobic induction
XP_010109903.1	880	26	essential for the anaerobic induction
XP_010109903.1	1419	26	essential for the anaerobic induction
XP_010109903.1	1040	26	light responsiveness
XP_010109903.1	1740	26	light responsiveness
XP_010109903.1	1027	27	gibberellin-responsive
XP_010109903.1	976	27	gibberellin-responsive
XP_010109903.1	1653	27	gibberellin-responsive
XP_010109903.1	1684	27	gibberellin-responsive
XP_010109903.1	273	27	light responsive
XP_010109903.1	274	26	light responsive
XP_010109903.1	921	26	MYB binding site involved in drought-inducibility
XP_010109903.1	1706	26	MYB binding site involved in drought-inducibility
XP_010109903.1	1466	29	part of a conserved DNA module involved in light responsiveness
XP_010109903.1	242	26	part of a conserved DNA module involved in light responsiveness

XP_010109903.1	248	26	part of a conserved DNA module involved in light responsiveness
XP_010109903.1	505	26	part of a conserved DNA module involved in light responsiveness
XP_010109903.1	623	26	part of a conserved DNA module involved in light responsiveness
XP_010109903.1	696	28	part of a light responsive element
XP_010109903.1	1450	28	part of a light responsive element
XP_010109903.1	1293	27	part of a light responsive element
XP_010109903.1	110	26	part of a light responsive element
XP_010109903.1	512	26	part of a light responsive element
XP_010109903.1	1364	26	part of a light responsive element
XP_010112575.1	713	29	involved in salicylic acid responsiveness
XP_010112575.1	718	29	involved in salicylic acid responsiveness
XP_010112575.1	61	25	involved in the abscisic acid responsiveness
XP_010112575.1	1567	25	involved in the abscisic acid responsiveness
XP_010112575.1	1829	25	involved in the abscisic acid responsiveness
XP_010112575.1	61	26	light responsiveness
XP_010112575.1	1829	26	light responsiveness
XP_010112575.1	1566	26	light responsiveness
XP_010112575.1	729	25	involved in the MeJA-responsiveness
XP_010112575.1	729	25	involved in the MeJA-responsiveness
XP_010112575.1	52	27	involved in endosperm expression
XP_010112575.1	119	27	gibberellin-responsive
XP_010112575.1	696	27	gibberellin-responsive
XP_010112575.1	1932	30	part of a conserved DNA module involved in light responsiveness
XP_010112575.1	411	28	part of a light responsive element
XP_010112575.1	40	28	part of a light responsive element
XP_010112575.1	1815	27	part of a light responsive element

XP_010112575.1	1938	30	part of a light responsive element
XP_010112575.1	368	26	part of a light responsive element
XP_010112575.1	851	26	part of a light responsive element
XP_010112575.1	1149	26	part of a light responsive element
XP_010112647.2	313	26	auxin-responsive
XP_010112647.2	757	26	auxin-responsive
XP_010112647.2	1265	29	involved in defense and stress responsiveness
XP_010112647.2	215	27	involved in the abscisic acid responsiveness
XP_010112647.2	826	29	involved in the abscisic acid responsiveness
XP_010112647.2	486	26	essential for the anaerobic induction
XP_010112647.2	625	26	essential for the anaerobic induction
XP_010112647.2	413	28	involved in differentiation of the palisade mesophyll cells
XP_010112647.2	1027	26	light responsive
XP_010112647.2	477	26	MYB binding site involved in drought-inducibility
XP_010112647.2	1074	26	part of a conserved DNA module involved in light responsiveness
XP_010112647.2	763	28	part of a light responsive element
XP_010112647.2	505	30	part of a light responsive element
XP_024017058.1	1319	29	involved in defense and stress responsiveness
XP_024017058.1	1634	30	involved in defense and stress responsiveness
XP_024017058.1	287	25	involved in the abscisic acid responsiveness
XP_024017058.1	346	25	involved in the abscisic acid responsiveness
XP_024017058.1	795	25	involved in the abscisic acid responsiveness
XP_024017058.1	1189	26	essential for the anaerobic induction
XP_024017058.1	1351	26	essential for the anaerobic induction
XP_024017058.1	1473	26	essential for the anaerobic induction
XP_024017058.1	1478	26	essential for the anaerobic induction

XP_024017058.1	1695	26	essential for the anaerobic induction
XP_024017058.1	286	26	light responsiveness
XP_024017058.1	346	26	light responsiveness
XP_024017058.1	794	26	light responsiveness
XP_024017058.1	749	25	involved in the MeJA-responsiveness
XP_024017058.1	1060	25	involved in the MeJA-responsiveness
XP_024017058.1	1515	25	involved in the MeJA-responsiveness
XP_024017058.1	749	25	involved in the MeJA-responsiveness
XP_024017058.1	1060	25	involved in the MeJA-responsiveness
XP_024017058.1	1515	25	involved in the MeJA-responsiveness
XP_024017058.1	887	26	related to meristem expression
XP_024017058.1	400	27	gibberellin-responsive
XP_024017058.1	992	27	gibberellin-responsive
XP_024017058.1	1193	26	MYB binding site involved in drought-inducibility
XP_024017058.1	50	31	MYB binding site involved in flavonoid biosynthetic genes regulation
XP_024017058.1	1420	27	MYB binding site involved in light responsiveness
XP_024017058.1	459	26	part of a conserved DNA module involved in light responsiveness
XP_024017058.1	669	26	part of a conserved DNA module involved in light responsiveness
XP_024017058.1	753	26	part of a conserved DNA module involved in light responsiveness
XP_024017058.1	1238	26	part of a conserved DNA module involved in light responsiveness
XP_024017058.1	1813	26	part of a conserved DNA module involved in light responsiveness
XP_024017058.1	1258	29	part of a light responsive element
XP_024017058.1	1711	29	part of a light responsive element
XP_024017058.1	1573	26	part of a light responsive element
XP_024017058.1	1638	26	part of a light responsive element
XP_024017058.1	430	28	part of a module for light response

XP_024019354.1	1781	29	involved in cell cycle regulation
XP_024019354.1	1800	29	involved in cell cycle regulation
XP_024019354.1	1105	25	involved in the abscisic acid responsiveness
XP_024019354.1	1790	26	essential for the anaerobic induction
XP_024019354.1	1105	26	light responsiveness
XP_024019354.1	1543	26	light responsiveness
XP_024019354.1	1777	26	light responsiveness
XP_024019354.1	512	26	light responsive
XP_024019354.1	761	26	light responsive
XP_024019354.1	1716	27	MYB binding site involved in light responsiveness
XP_024019354.1	1752	27	MYB binding site involved in light responsiveness
XP_024019354.1	1782	26	MYBHv1 binding site
XP_024019354.1	1802	26	MYBHv1 binding site
XP_024019354.1	356	26	part of a conserved DNA module involved in light responsiveness
XP_024019354.1	607	26	part of a conserved DNA module involved in light responsiveness
XP_024019354.1	1275	26	part of a conserved DNA module involved in light responsiveness
XP_024019354.1	1899	29	part of a light responsive element
XP_024019354.1	345	33	part of a light responsive module
XP_024019354.1	1022	33	part of a light responsive module
XP_024019354.1	1471	28	part of a module for light response
XP_024020016.1	1431	26	involved in low-temperature responsiveness
XP_024020016.1	117	29	involved in the abscisic acid responsiveness
XP_024020016.1	888	25	involved in the abscisic acid responsiveness
XP_024020016.1	759	26	essential for the anaerobic induction
XP_024020016.1	780	26	essential for the anaerobic induction
XP_024020016.1	1215	26	essential for the anaerobic induction

XP_024020016.1	1387	26	essential for the anaerobic induction
XP_024020016.1	1517	26	essential for the anaerobic induction
XP_024020016.1	888	26	light responsiveness
XP_024020016.1	104	29	involved in zein metabolism regulation
XP_024020016.1	170	27	gibberellin-responsive
XP_024020016.1	973	26	light responsive
XP_024020016.1	392	26	MYB binding site involved in drought-inducibility
XP_024020016.1	219	26	part of a conserved DNA module involved in light responsiveness
XP_024020016.1	279	26	part of a conserved DNA module involved in light responsiveness
XP_024020016.1	285	26	part of a conserved DNA module involved in light responsiveness
XP_024020016.1	765	26	part of a conserved DNA module involved in light responsiveness
XP_024020016.1	961	26	part of a conserved DNA module involved in light responsiveness
XP_024020016.1	1047	26	part of a conserved DNA module involved in light responsiveness
XP_024020016.1	1263	26	part of a conserved DNA module involved in light responsiveness
XP_024020016.1	1717	27	part of a light responsive element
XP_024020016.1	1810	27	part of a light responsive element
XP_024020016.1	1863	27	part of a light responsive element
XP_024020016.1	1284	33	part of a light responsive module
XP_024020016.1	544	28	part of a module for light response
XP_024020043.1	284	26	involved in low-temperature responsiveness
XP_024020043.1	379	26	involved in low-temperature responsiveness
XP_024020043.1	506	25	involved in the abscisic acid responsiveness
XP_024020043.1	532	25	involved in the abscisic acid responsiveness
XP_024020043.1	292	26	essential for the anaerobic induction
XP_024020043.1	545	26	essential for the anaerobic induction
XP_024020043.1	1012	26	essential for the anaerobic induction

XP_024020043.1	506	26	light responsiveness
XP_024020043.1	531	26	light responsiveness
XP_024020043.1	530	25	involved in the MeJA-responsiveness
XP_024020043.1	530	25	involved in the MeJA-responsiveness
XP_024020043.1	1372	26	related to meristem expression
XP_024020043.1	70	27	involved in endosperm expression
XP_024020043.1	1090	27	involved in endosperm expression
XP_024020043.1	1028	26	involved in anoxic specific inducibility
XP_024020043.1	367	26	light responsive
XP_024020043.1	871	26	MYBHv1 binding site
XP_024020043.1	225	29	part of a conserved DNA module involved in light responsiveness
XP_024020043.1	34	26	part of a conserved DNA module involved in light responsiveness
XP_024020043.1	667	26	part of a conserved DNA module involved in light responsiveness
XP_024020043.1	687	26	part of a conserved DNA module involved in light responsiveness
XP_024020043.1	1292	26	part of a conserved DNA module involved in light responsiveness
XP_024020043.1	116	27	part of a light responsive element
XP_024020043.1	145	27	part of a light responsive element
XP_024020043.1	174	29	part of a light responsive element
XP_024020043.1	1246	30	part of a light responsive element
XP_024020043.1	1246	30	part of a light responsive element
XP_024020043.1	1286	26	part of a light responsive element
XP_024020043.1	850	28	part of a module for light response
XP_024022630.1	1507	26	involved in low-temperature responsiveness
XP_024022630.1	1425	29	involved in salicylic acid responsiveness
XP_024022630.1	110	25	involved in the abscisic acid responsiveness
XP_024022630.1	1322	25	involved in the abscisic acid responsiveness

XP_024022630.1	797	26	essential for the anaerobic induction
XP_024022630.1	852	26	essential for the anaerobic induction
XP_024022630.1	1131	26	essential for the anaerobic induction
XP_024022630.1	1720	27	involved in auxin responsiveness
XP_024022630.1	109	26	light responsiveness
XP_024022630.1	1729	26	light responsiveness
XP_024022630.1	1322	26	light responsiveness
XP_024022630.1	1000	25	involved in the MeJA-responsiveness
XP_024022630.1	1000	25	involved in the MeJA-responsiveness
XP_024022630.1	594	29	involved in zein metabolism regulation
XP_024022630.1	1533	26	related to meristem expression
XP_024022630.1	504	27	gibberellin-responsive
XP_024022630.1	328	27	gibberellin-responsive
XP_024022630.1	1685	26	light responsive
XP_024022630.1	785	26	MYB binding site involved in drought-inducibility
XP_024022630.1	600	26	MYBHv1 binding site
XP_024022630.1	156	26	part of a conserved DNA module involved in light responsiveness
XP_024022630.1	1360	26	part of a conserved DNA module involved in light responsiveness
XP_024022630.1	1519	26	part of a conserved DNA module involved in light responsiveness
XP_024022630.1	1677	26	part of a conserved DNA module involved in light responsiveness
XP_024022630.1	124	28	part of a module for light response
XP_024022632.1	1724	29	involved in light responsiveness
XP_024022632.1	940	29	involved in salicylic acid responsiveness
XP_024022632.1	269	26	involved in the abscisic acid responsiveness
XP_024022632.1	270	25	involved in the abscisic acid responsiveness
XP_024022632.1	290	26	involved in the abscisic acid responsiveness

XP_024022632.1	291	25	involved in the abscisic acid responsiveness
XP_024022632.1	1591	25	involved in the abscisic acid responsiveness
XP_024022632.1	584	26	essential for the anaerobic induction
XP_024022632.1	1573	26	essential for the anaerobic induction
XP_024022632.1	267	29	light responsiveness
XP_024022632.1	269	26	light responsiveness
XP_024022632.1	290	26	light responsiveness
XP_024022632.1	1591	26	light responsiveness
XP_024022632.1	269	26	light responsiveness
XP_024022632.1	290	26	light responsiveness
XP_024022632.1	1598	28	involved in seed-specific regulation
XP_024022632.1	1699	28	involved in seed-specific regulation
XP_024022632.1	566	25	involved in the MeJA-responsiveness
XP_024022632.1	1149	25	involved in the MeJA-responsiveness
XP_024022632.1	1593	25	involved in the MeJA-responsiveness
XP_024022632.1	1690	25	involved in the MeJA-responsiveness
XP_024022632.1	566	25	involved in the MeJA-responsiveness
XP_024022632.1	1149	25	involved in the MeJA-responsiveness
XP_024022632.1	1593	25	involved in the MeJA-responsiveness
XP_024022632.1	1690	25	involved in the MeJA-responsiveness
XP_024022632.1	289	29	involved in zein metabolism regulation
XP_024022632.1	1836	29	involved in zein metabolism regulation
XP_024022632.1	1240	26	related to meristem expression
XP_024022632.1	1438	26	related to meristem expression
XP_024022632.1	1447	26	related to meristem expression
XP_024022632.1	1503	29	part of a conserved DNA module involved in light responsiveness

XP_024022632.1	1544	26	part of a conserved DNA module involved in light responsiveness
XP_024022632.1	1782	28	part of a light responsive element
XP_024022632.1	238	26	part of a light responsive element
XP_024022632.1	857	28	part of a module for light response
XP_024022632.1	650	31	protein binding site
XP_024022961.1	1035	26	auxin-responsive
XP_024022961.1	1364	26	auxin-responsive
XP_024022961.1	1547	29	involved in light responsiveness
XP_024022961.1	1635	29	involved in light responsiveness
XP_024022961.1	255	26	involved in low-temperature responsiveness
XP_024022961.1	1339	25	involved in the abscisic acid responsiveness
XP_024022961.1	1549	28	involved in the abscisic acid responsiveness
XP_024022961.1	1550	25	involved in the abscisic acid responsiveness
XP_024022961.1	1574	25	involved in the abscisic acid responsiveness
XP_024022961.1	1617	25	involved in the abscisic acid responsiveness
XP_024022961.1	1634	31	involved in the abscisic acid responsiveness
XP_024022961.1	1635	29	involved in the abscisic acid responsiveness
XP_024022961.1	1637	28	involved in the abscisic acid responsiveness
XP_024022961.1	1638	25	involved in the abscisic acid responsiveness
XP_024022961.1	1549	26	light responsiveness
XP_024022961.1	1574	26	light responsiveness
XP_024022961.1	1637	26	light responsiveness
XP_024022961.1	1339	26	light responsiveness
XP_024022961.1	1616	26	light responsiveness
XP_024022961.1	76	25	involved in the MeJA-responsiveness
XP_024022961.1	502	25	involved in the MeJA-responsiveness

XP_024022961.1	1055	25	involved in the MeJA-responsiveness
XP_024022961.1	1431	25	involved in the MeJA-responsiveness
XP_024022961.1	1576	25	involved in the MeJA-responsiveness
XP_024022961.1	1632	25	involved in the MeJA-responsiveness
XP_024022961.1	76	25	involved in the MeJA-responsiveness
XP_024022961.1	502	25	involved in the MeJA-responsiveness
XP_024022961.1	1055	25	involved in the MeJA-responsiveness
XP_024022961.1	1431	25	involved in the MeJA-responsiveness
XP_024022961.1	1576	25	involved in the MeJA-responsiveness
XP_024022961.1	1632	25	involved in the MeJA-responsiveness
XP_024022961.1	683	27	gibberellin-responsive
XP_024022961.1	-7	26	light responsive
XP_024022961.1	796	26	light responsive
XP_024022961.1	569	29	part of a conserved DNA module involved in light responsiveness
XP_024022961.1	860	26	part of a conserved DNA module involved in light responsiveness
XP_024022961.1	901	26	part of a conserved DNA module involved in light responsiveness
XP_024022961.1	1665	27	part of a light responsive element
XP_024022961.1	616	28	part of a light responsive element
XP_024023006.1	416	30	ATBP-1
XP_024023006.1	333	25	involved in the abscisic acid responsiveness
XP_024023006.1	553	25	involved in the abscisic acid responsiveness
XP_024023006.1	980	27	involved in the abscisic acid responsiveness
XP_024023006.1	1188	25	involved in the abscisic acid responsiveness
XP_024023006.1	1214	26	involved in the abscisic acid responsiveness
XP_024023006.1	1215	25	involved in the abscisic acid responsiveness
XP_024023006.1	849	26	essential for the anaerobic induction

XP_024023006.1	964	26	essential for the anaerobic induction
XP_024023006.1	333	26	light responsiveness
XP_024023006.1	1188	26	light responsiveness
XP_024023006.1	1214	26	light responsiveness
XP_024023006.1	552	26	light responsiveness
XP_024023006.1	1214	26	light responsiveness
XP_024023006.1	335	25	involved in the MeJA-responsiveness
XP_024023006.1	1553	25	involved in the MeJA-responsiveness
XP_024023006.1	335	25	involved in the MeJA-responsiveness
XP_024023006.1	1553	25	involved in the MeJA-responsiveness
XP_024023006.1	116	26	MYB binding site involved in drought-inducibility
XP_024023006.1	1073	27	MYB binding site involved in light responsiveness
XP_024023006.1	1557	29	part of a conserved DNA module involved in light responsiveness
XP_024023006.1	855	26	part of a conserved DNA module involved in light responsiveness
XP_024023006.1	1001	26	part of a conserved DNA module involved in light responsiveness
XP_024023006.1	1433	26	part of a conserved DNA module involved in light responsiveness
XP_024023006.1	1437	26	part of a conserved DNA module involved in light responsiveness
XP_024023006.1	1650	26	part of a conserved DNA module involved in light responsiveness
XP_024023006.1	694	28	part of a light responsive element
XP_024023006.1	1883	29	part of a light responsive element
XP_024023006.1	593	29	part of a light responsive element
XP_024023006.1	1611	27	part of a light responsive element
XP_024023006.1	1121	26	part of a light responsive element
XP_024023006.1	958	28	part of a module for light response
XP_024023006.1	373	32	part of gapA in (gapA-CMA1) involved with light responsiveness
XP_024023382.1	444	30	ATBP-1

XP_024023382.1	1123	29	involved in light responsiveness
XP_024023382.1	59	29	involved in salicylic acid responsiveness
XP_024023382.1	255	29	involved in salicylic acid responsiveness
XP_024023382.1	503	29	involved in salicylic acid responsiveness
XP_024023382.1	352	25	involved in the abscisic acid responsiveness
XP_024023382.1	1125	25	involved in the abscisic acid responsiveness
XP_024023382.1	523	26	essential for the anaerobic induction
XP_024023382.1	352	26	light responsiveness
XP_024023382.1	1125	26	light responsiveness
XP_024023382.1	829	28	involved in seed-specific regulation
XP_024023382.1	1050	25	involved in the MeJA-responsiveness
XP_024023382.1	1050	25	involved in the MeJA-responsiveness
XP_024023382.1	1770	29	maximal elicitor-mediated activation (2copies)
XP_024023382.1	1300	26	MYB binding site involved in drought-inducibility
XP_024023382.1	563	26	part of a conserved DNA module involved in light responsiveness
XP_024023382.1	851	26	part of a conserved DNA module involved in light responsiveness
XP_024023382.1	855	26	part of a conserved DNA module involved in light responsiveness
XP_024023382.1	1010	26	part of a conserved DNA module involved in light responsiveness
XP_024023382.1	422	30	part of a light responsive element
XP_024023382.1	162	26	part of a light responsive element
XP_024023382.1	1309	26	part of a light responsive element
XP_024023462.1	1580	26	auxin-responsive
XP_024023462.1	1533	29	involved in defense and stress responsiveness
XP_024023462.1	1702	29	involved in defense and stress responsiveness
XP_024023462.1	1912	26	involved in low-temperature responsiveness
XP_024023462.1	610	29	involved in salicylic acid responsiveness

XP_024023462.1	1347	25	involved in the abscisic acid responsiveness
XP_024023462.1	1392	25	involved in the abscisic acid responsiveness
XP_024023462.1	1796	27	involved in the abscisic acid responsiveness
XP_024023462.1	358	26	essential for the anaerobic induction
XP_024023462.1	1440	26	essential for the anaerobic induction
XP_024023462.1	1176	26	light responsiveness
XP_024023462.1	1346	28	light responsiveness
XP_024023462.1	1347	26	light responsiveness
XP_024023462.1	1375	26	light responsiveness
XP_024023462.1	1392	26	light responsiveness
XP_024023462.1	1712	25	involved in the MeJA-responsiveness
XP_024023462.1	1712	25	involved in the MeJA-responsiveness
XP_024023462.1	801	26	light responsive
XP_024023462.1	1104	27	light responsive
XP_024023462.1	1672	26	MYBHv1 binding site
XP_024023462.1	587	29	part of a conserved DNA module involved in light responsiveness
XP_024023462.1	23	30	part of a conserved DNA module involved in light responsiveness
XP_024023462.1	386	26	part of a conserved DNA module involved in light responsiveness
XP_024023462.1	820	26	part of a conserved DNA module involved in light responsiveness
XP_024023462.1	1082	26	part of a conserved DNA module involved in light responsiveness
XP_024023462.1	1114	26	part of a conserved DNA module involved in light responsiveness
XP_024023462.1	1123	26	part of a conserved DNA module involved in light responsiveness
XP_024023462.1	1187	26	part of a conserved DNA module involved in light responsiveness
XP_024023462.1	1206	26	part of a conserved DNA module involved in light responsiveness
XP_024023462.1	1777	26	part of a conserved DNA module involved in light responsiveness
XP_024023462.1	1892	30	part of a light responsive element

XP_024023501.1	957	29	involved in salicylic acid responsiveness
XP_024023501.1	1506	29	involved in salicylic acid responsiveness
XP_024023501.1	1806	29	involved in salicylic acid responsiveness
XP_024023501.1	501	25	involved in the abscisic acid responsiveness
XP_024023501.1	1886	25	involved in the abscisic acid responsiveness
XP_024023501.1	145	26	essential for the anaerobic induction
XP_024023501.1	326	26	essential for the anaerobic induction
XP_024023501.1	374	26	essential for the anaerobic induction
XP_024023501.1	1021	26	essential for the anaerobic induction
XP_024023501.1	1062	26	essential for the anaerobic induction
XP_024023501.1	1456	26	essential for the anaerobic induction
XP_024023501.1	500	26	light responsiveness
XP_024023501.1	1885	26	light responsiveness
XP_024023501.1	1651	28.5	involved in zein metabolism regulation
XP_024023501.1	139	26	related to meristem expression
XP_024023501.1	1424	27	gibberellin-responsive
XP_024023501.1	-1	26	light responsive
XP_024023501.1	1523	29	light responsive
XP_024023501.1	1687	30.5	MYB binding site involved in flavonoid biosynthetic genes regulation
XP_024023501.1	1463	26	MYBHv1 binding site
XP_024023501.1	245	26	part of a conserved DNA module involved in light responsiveness
XP_024023501.1	361	26	part of a conserved DNA module involved in light responsiveness
XP_024023501.1	1725	26	part of a conserved DNA module involved in light responsiveness
XP_024023501.1	47	29	part of a light responsive element
XP_024023501.1	666	27	part of a light responsive element
XP_024023501.1	724	26	part of a light responsive element

XP_024023501.1	314	28	part of a module for light response
XP_024024700.1	1477	26	involved in low-temperature responsiveness
XP_024024700.1	1263	25	involved in the abscisic acid responsiveness
XP_024024700.1	1682	26	essential for the anaerobic induction
XP_024024700.1	1263	26	light responsiveness
XP_024024700.1	1888	26	light responsiveness
XP_024024700.1	320	25	involved in the MeJA-responsiveness
XP_024024700.1	1265	25	involved in the MeJA-responsiveness
XP_024024700.1	1291	25	involved in the MeJA-responsiveness
XP_024024700.1	320	25	involved in the MeJA-responsiveness
XP_024024700.1	1265	25	involved in the MeJA-responsiveness
XP_024024700.1	1291	25	involved in the MeJA-responsiveness
XP_024024700.1	816	29	involved in zein metabolism regulation
XP_024024700.1	1848	29	involved in zein metabolism regulation
XP_024024700.1	745	26	related to meristem expression
XP_024024700.1	433	26	light responsive
XP_024024700.1	477	26	part of a conserved DNA module involved in light responsiveness
XP_024024700.1	1486	26	part of a conserved DNA module involved in light responsiveness
XP_024024700.1	1352	27	part of a light responsive element
XP_024024700.1	529	28	part of a light responsive element
XP_024024700.1	1257	28	part of a light responsive element
XP_024024700.1	1169	27	part of a light responsive element
XP_024024700.1	988	26	part of a light responsive element
XP_024024700.1	1368	26	part of a light responsive element
XP_024024700.1	1789	31	protein binding site
XP_024024700.1	442	29	wound-responsive element

XP_024025182.1	56	29	involved in defense and stress responsiveness
XP_024025182.1	589	25	involved in the abscisic acid responsiveness
XP_024025182.1	1814	25	involved in the abscisic acid responsiveness
XP_024025182.1	648	26	essential for the anaerobic induction
XP_024025182.1	759	26	essential for the anaerobic induction
XP_024025182.1	1571	26	essential for the anaerobic induction
XP_024025182.1	1867	26	essential for the anaerobic induction
XP_024025182.1	373	29	involved in circadian control
XP_024025182.1	221	26	light responsiveness
XP_024025182.1	586	28	light responsiveness
XP_024025182.1	1687	26	light responsiveness
XP_024025182.1	1813	26	light responsiveness
XP_024025182.1	588	26	light responsiveness
XP_024025182.1	176	25	involved in the MeJA-responsiveness
XP_024025182.1	176	25	involved in the MeJA-responsiveness
XP_024025182.1	93	28	involved in zein metabolism regulation
XP_024025182.1	891	29	involved in zein metabolism regulation
XP_024025182.1	1967	27	involved in endosperm expression
XP_024025182.1	616	27	gibberellin-responsive
XP_024025182.1	645	30.5	MYB binding site involved in flavonoid biosynthetic genes regulation
XP_024025182.1	-10	26	part of a conserved DNA module involved in light responsiveness
XP_024025182.1	315	26	part of a conserved DNA module involved in light responsiveness
XP_024025182.1	666	26	part of a conserved DNA module involved in light responsiveness
XP_024025182.1	712	28	part of a light responsive element
XP_024025182.1	1803	30	part of a light responsive element
XP_024025182.1	1772	27	part of a light responsive element

XP_024025676.1	1641	30	involved in defense and stress responsiveness
XP_024025676.1	285	25	involved in the abscisic acid responsiveness
XP_024025676.1	344	25	involved in the abscisic acid responsiveness
XP_024025676.1	793	25	involved in the abscisic acid responsiveness
XP_024025676.1	1480	26	essential for the anaerobic induction
XP_024025676.1	1485	26	essential for the anaerobic induction
XP_024025676.1	1702	26	essential for the anaerobic induction
XP_024025676.1	284	26	light responsiveness
XP_024025676.1	344	26	light responsiveness
XP_024025676.1	792	26	light responsiveness
XP_024025676.1	747	25	involved in the MeJA-responsiveness
XP_024025676.1	1058	25	involved in the MeJA-responsiveness
XP_024025676.1	1522	25	involved in the MeJA-responsiveness
XP_024025676.1	747	25	involved in the MeJA-responsiveness
XP_024025676.1	1058	25	involved in the MeJA-responsiveness
XP_024025676.1	1522	25	involved in the MeJA-responsiveness
XP_024025676.1	885	26	related to meristem expression
XP_024025676.1	398	27	gibberellin-responsive
XP_024025676.1	990	27	gibberellin-responsive
XP_024025676.1	50	31	MYB binding site involved in flavonoid biosynthetic genes regulation
XP_024025676.1	1427	27	MYB binding site involved in light responsiveness
XP_024025676.1	457	26	part of a conserved DNA module involved in light responsiveness
XP_024025676.1	667	26	part of a conserved DNA module involved in light responsiveness
XP_024025676.1	751	26	part of a conserved DNA module involved in light responsiveness
XP_024025676.1	1236	26	part of a conserved DNA module involved in light responsiveness
XP_024025676.1	1820	26	part of a conserved DNA module involved in light responsiveness

XP_024025676.1	1256	29	part of a light responsive element
XP_024025676.1	1718	29	part of a light responsive element
XP_024025676.1	1580	26	part of a light responsive element
XP_024025676.1	1645	26	part of a light responsive element
XP_024025676.1	428	28	part of a module for light response
XP_024026054.1	1141	29	involved in defense and stress responsiveness
XP_024026054.1	217	25	involved in the abscisic acid responsiveness
XP_024026054.1	1329	25	involved in the abscisic acid responsiveness
XP_024026054.1	84	26	essential for the anaerobic induction
XP_024026054.1	105	26	essential for the anaerobic induction
XP_024026054.1	313	26	essential for the anaerobic induction
XP_024026054.1	362	26	essential for the anaerobic induction
XP_024026054.1	1447	26	essential for the anaerobic induction
XP_024026054.1	217	26	light responsiveness
XP_024026054.1	1328	26	light responsiveness
XP_024026054.1	592	25	involved in the MeJA-responsiveness
XP_024026054.1	592	25	involved in the MeJA-responsiveness
XP_024026054.1	1116	27	involved in endosperm expression
XP_024026054.1	403	27	gibberellin-responsive
XP_024026054.1	10	27	gibberellin-responsive
XP_024026054.1	1226	26	light responsive
XP_024026054.1	1887	26	light responsive
XP_024026054.1	1884	27	MYB binding site involved in light responsiveness
XP_024026054.1	1172	29	part of a conserved DNA module involved in light responsiveness
XP_024026054.1	90	26	part of a conserved DNA module involved in light responsiveness
XP_024026054.1	378	26	part of a conserved DNA module involved in light responsiveness

XP_024026054.1	382	26	part of a conserved DNA module involved in light responsiveness
XP_024026054.1	584	26	part of a conserved DNA module involved in light responsiveness
XP_024026054.1	920	26	part of a conserved DNA module involved in light responsiveness
XP_024026054.1	1179	26	part of a conserved DNA module involved in light responsiveness
XP_024026054.1	1700	26	part of a conserved DNA module involved in light responsiveness
XP_024026054.1	1796	26	part of a conserved DNA module involved in light responsiveness
XP_024026054.1	1660	28	part of a light responsive element
XP_024026054.1	1679	28	part of a light responsive element
XP_024026054.1	1170	29	part of a light responsive element
XP_024026054.1	1171	30	part of a light responsive element
XP_024026054.1	1452	30	part of a light responsive element
XP_024026054.1	1490	29	part of a light responsive element
XP_024026054.1	1492	30	part of a light responsive element
XP_024026054.1	1491	29	part of a light responsive element
XP_024026054.1	1141	26	part of a light responsive element
XP_024026054.1	1459	26	part of a light responsive element
XP_024026054.1	1389	28	part of a module for light response
XP_024026540.1	903	26	auxin-responsive
XP_024026540.1	1074	26	auxin-responsive
XP_024026540.1	1466	26	auxin-responsive
XP_024026540.1	242	29	involved in cell cycle regulation
XP_024026540.1	1813	29	involved in defense and stress responsiveness
XP_024026540.1	207	27	involved in gibberellin-responsiveness
XP_024026540.1	1208	29	involved in light responsiveness
XP_024026540.1	1471	26	involved in low-temperature responsiveness
XP_024026540.1	879	29	involved in salicylic acid responsiveness

XP_024026540.1	222	25	involved in the abscisic acid responsiveness
XP_024026540.1	299	27	involved in the abscisic acid responsiveness
XP_024026540.1	713	26	involved in the abscisic acid responsiveness
XP_024026540.1	714	25	involved in the abscisic acid responsiveness
XP_024026540.1	1823	26	essential for the anaerobic induction
XP_024026540.1	713	26	light responsiveness
XP_024026540.1	222	26	light responsiveness
XP_024026540.1	711	29	light responsiveness
XP_024026540.1	713	26	light responsiveness
XP_024026540.1	401	25	involved in the MeJA-responsiveness
XP_024026540.1	404	25	involved in the MeJA-responsiveness
XP_024026540.1	1050	25	involved in the MeJA-responsiveness
XP_024026540.1	1263	25	involved in the MeJA-responsiveness
XP_024026540.1	1882	25	involved in the MeJA-responsiveness
XP_024026540.1	401	25	involved in the MeJA-responsiveness
XP_024026540.1	404	25	involved in the MeJA-responsiveness
XP_024026540.1	1050	25	involved in the MeJA-responsiveness
XP_024026540.1	1263	25	involved in the MeJA-responsiveness
XP_024026540.1	1882	25	involved in the MeJA-responsiveness
XP_024026540.1	792	27	involved in endosperm expression
XP_024026540.1	122	31	involved in endosperm-specific negative expression
XP_024026540.1	1029	26	MYB binding site involved in drought-inducibility
XP_024026540.1	243	26	MYBHv1 binding site
XP_024026540.1	689	26	MYBHv1 binding site
XP_024026540.1	711	29	part of a light responsive element
XP_024026540.1	737	30	part of a light responsive element

XP_024026540.1	1492	29	part of a light responsive element
XP_024026540.1	887	29	wound-responsive element
XP_024030012.1	1169	29	involved in defense and stress responsiveness
XP_024030012.1	10	26	involved in low-temperature responsiveness
XP_024030012.1	143	29	involved in salicylic acid responsiveness
XP_024030012.1	899	29	involved in salicylic acid responsiveness
XP_024030012.1	1221	29	involved in salicylic acid responsiveness
XP_024030012.1	531	25	involved in the abscisic acid responsiveness
XP_024030012.1	531	26	light responsiveness
XP_024030012.1	1643	29	part of a conserved DNA module involved in light responsiveness
XP_024030012.1	15	26	part of a conserved DNA module involved in light responsiveness
XP_024030012.1	91	26	part of a conserved DNA module involved in light responsiveness
XP_024030012.1	184	26	part of a conserved DNA module involved in light responsiveness
XP_024030012.1	925	26	part of a conserved DNA module involved in light responsiveness
XP_024030012.1	1411	26	part of a conserved DNA module involved in light responsiveness
XP_024030012.1	1670	26	part of a conserved DNA module involved in light responsiveness
XP_024030012.1	1784	26	part of a conserved DNA module involved in light responsiveness
XP_024030012.1	346	29.5	part of a light responsive element
XP_024030012.1	212	29	part of a light responsive element
XP_024030012.1	1248	27	part of a light responsive element
XP_024030012.1	1953	27	part of a light responsive element
XP_024030012.1	1265	26	part of a light responsive element
XP_024030227.1	1806	29	involved in cell cycle regulation
XP_024030227.1	1445	29	involved in defense and stress responsiveness
XP_024030227.1	1219	27	involved in gibberellin-responsiveness
XP_024030227.1	1240	27	involved in gibberellin-responsiveness

XP_024030227.1	64	25	involved in the abscisic acid responsiveness
XP_024030227.1	446	25	involved in the abscisic acid responsiveness
XP_024030227.1	779	25	involved in the abscisic acid responsiveness
XP_024030227.1	999	25	involved in the abscisic acid responsiveness
XP_024030227.1	1043	27	involved in the abscisic acid responsiveness
XP_024030227.1	1540	25	involved in the abscisic acid responsiveness
XP_024030227.1	63	26	light responsiveness
XP_024030227.1	446	26	light responsiveness
XP_024030227.1	779	26	light responsiveness
XP_024030227.1	999	26	light responsiveness
XP_024030227.1	1539	26	light responsiveness
XP_024030227.1	32	25	involved in the MeJA-responsiveness
XP_024030227.1	216	25	involved in the MeJA-responsiveness
XP_024030227.1	1001	25	involved in the MeJA-responsiveness
XP_024030227.1	1595	25	involved in the MeJA-responsiveness
XP_024030227.1	32	25	involved in the MeJA-responsiveness
XP_024030227.1	216	25	involved in the MeJA-responsiveness
XP_024030227.1	1001	25	involved in the MeJA-responsiveness
XP_024030227.1	1595	25	involved in the MeJA-responsiveness
XP_024030227.1	427	27	gibberellin-responsive
XP_024030227.1	1645	27	gibberellin-responsive
XP_024030227.1	1019	26	light responsive
XP_024030227.1	1415	27	light responsive
XP_024030227.1	1416	26	light responsive
XP_024030227.1	1175	30.5	MYB binding site involved in flavonoid biosynthetic genes regulation
XP_024030227.1	1417	32	MYB binding site involved in flavonoid biosynthetic genes regulation

XP_024030227.1	1808	26	MYBHv1 binding site
XP_024030227.1	413	26	part of a conserved DNA module involved in light responsiveness
XP_024030227.1	724	26	part of a conserved DNA module involved in light responsiveness
XP_024030227.1	1469	26	part of a conserved DNA module involved in light responsiveness
XP_024030227.1	1899	26	part of a conserved DNA module involved in light responsiveness
XP_024030227.1	1913	26	part of a conserved DNA module involved in light responsiveness
XP_024030227.1	598	28	part of a light responsive element
XP_024030227.1	1227	30	part of a light responsive element
XP_024030227.1	121	30	part of a light responsive element
XP_024031300.1	503	29	involved in light responsiveness
XP_024031300.1	1102	26	involved in low-temperature responsiveness
XP_024031300.1	3	26	involved in the abscisic acid responsiveness
XP_024031300.1	4	25	involved in the abscisic acid responsiveness
XP_024031300.1	763	26	essential for the anaerobic induction
XP_024031300.1	818	26	essential for the anaerobic induction
XP_024031300.1	1928	26	essential for the anaerobic induction
XP_024031300.1	3	26	light responsiveness
XP_024031300.1	3	26	light responsiveness
XP_024031300.1	1874	27	gibberellin-responsive
XP_024031300.1	1135	27	gibberellin-responsive
XP_024031300.1	574	26	light responsive
XP_024031300.1	1778	26	MYB binding site involved in drought-inducibility
XP_024031300.1	640	27	MYB binding site involved in light responsiveness
XP_024031300.1	686	27	MYB binding site involved in light responsiveness
XP_024031300.1	1344	26	MYBHv1 binding site
XP_024031300.1	80	26	part of a conserved DNA module involved in light responsiveness

XP_024031300.1	146	26	part of a conserved DNA module involved in light responsiveness
XP_024031300.1	367	26	part of a conserved DNA module involved in light responsiveness
XP_024031300.1	952	26	part of a conserved DNA module involved in light responsiveness
XP_024031300.1	986	26	part of a conserved DNA module involved in light responsiveness
XP_024031300.1	596	29	part of a light responsive element
XP_024031300.1	1150	30	part of a light responsive element
XP_024031300.1	1215	30	part of a light responsive element
XP_024031300.1	597	29	part of a light responsive element
XP_024031300.1	598	29	part of a light responsive element
XP_024031300.1	713	28	part of a light responsive element
XP_024031300.1	1754	28	part of a light responsive element
XP_024031300.1	1295	27	part of a light responsive element
XP_024031300.1	553	26	part of a light responsive element
XP_024031300.1	1749	28	part of a module for light response
XP_024032012.1	207	30	ATBP-1
XP_024032012.1	973	30	ATBP-1
XP_024032012.1	1236	27	involved in gibberellin-responsiveness
XP_024032012.1	1667	26	involved in low-temperature responsiveness
XP_024032012.1	1366	29	involved in salicylic acid responsiveness
XP_024032012.1	284	26	involved in the abscisic acid responsiveness
XP_024032012.1	285	25	involved in the abscisic acid responsiveness
XP_024032012.1	848	30	involved in the abscisic acid responsiveness
XP_024032012.1	1825	25	involved in the abscisic acid responsiveness
XP_024032012.1	1160	26	essential for the anaerobic induction
XP_024032012.1	1358	26	essential for the anaerobic induction
XP_024032012.1	284	26	light responsiveness

XP_024032012.1	1824	26	light responsiveness
XP_024032012.1	284	26	light responsiveness
XP_024032012.1	1581	27	involved in endosperm expression
XP_024032012.1	876	28.5	involved in differentiation of the palisade mesophyll cells
XP_024032012.1	1882	28	involved in differentiation of the palisade mesophyll cells
XP_024032012.1	169	26	part of a conserved DNA module involved in light responsiveness
XP_024032012.1	1108	26	part of a conserved DNA module involved in light responsiveness
XP_024032012.1	1527	26	part of a conserved DNA module involved in light responsiveness
XP_024032012.1	1795	28	part of a light responsive element
XP_024032012.1	741	26	part of a light responsive element
XP_024032012.1	1482	28	part of a module for light response
XP_024032012.1	1708	29	wound-responsive element
XP_024032474.1	212	29	involved in light responsiveness
XP_024032474.1	212	29	involved in the abscisic acid responsiveness
XP_024032474.1	214	26	involved in the abscisic acid responsiveness
XP_024032474.1	215	25	involved in the abscisic acid responsiveness
XP_024032474.1	214	26	light responsiveness
XP_024032474.1	213	28	light responsiveness
XP_024032474.1	214	26	light responsiveness
XP_024032474.1	232	26	light responsiveness
XP_024032474.1	133	29	part of a light responsive element

Supplementary Table S3. Predicted transcription factors, matched sequence, binding sites in *Morus notabilis PLA* promoter regions.

Gene name	TF name	Starting position	Termination position	strand	matched sequence
XP_010101003.1	ATHB-16	96	103	+	TAATAATT
	ATHB-16	1554	1561	-	TAATAATT
	Dof2	1265	1269	+	AAAGC
	Dof2	1994	1998	-	AAAGC
	CDF2	562	568	+	AAAAAGT
XP_024022632.1	ARR11	1742	1749	+	AAGATACG
	PIF1	289	294	+	CACGTG
	PIF1	289	294	-	CACGTG
	PIF1	310	315	+	CACGTG
	PIF1	310	315	-	CACGTG
XP_010104276.2	Dof3	1730	1735	-	AAAGCG
	ABF3	470	476	+	ACGTGTC
	Dof2	1731	1735	-	AAAGC
	Dof2	1748	1752	+	AAAGC
	Dof2	1928	1932	-	AAAGC
XP_010104409.1	PIF1	683	688	+	CACGTG
	PIF1	683	688	-	CACGTG
	BEE2	683	688	+	CACGTG
	BEE2	683	688	-	CACGTG
	Dof2	703	707	-	AAAGC
XP_010090405.1	Dof3	1655	1660	-	AAAGCG
	CDF2	4	13	-	AAAAAAGTGA

	Dof2	71	75	-	AAAGC
	Dof2	1207	1211	+	AAAGC
	Dof2	1492	1496	-	AAAGC
XP_024025676.1	ABF3	813	819	+	ACGTGTC
	Dof2	641	645	-	AAAGC
	CDF2	1002	1008	-	AAAAAGT
	MaB1A	641	645	-	AAAGC
	PBF	641	645	-	AAAGC
XP_010112575.1	Dof3	580	585	-	AAAGCG
	ATHB-16	682	689	+	TAATAATT
	Dof2	289	293	+	AAAGC
	Dof2	581	585	-	AAAGC
	CDF2	1042	1048	+	AAAAAGT
XP_010109903.1	ATHB-16	1256	1263	+	TAATAATT
	Dof2	690	694	+	AAAGC
	Dof2	736	740	+	AAAGC
	Dof2	739	743	-	AAAGC
	MaB1A	690	694	+	AAAGC
XP_010105205.2	NAC058	1978	1985	+	ACACGCAA
	PIF1	1508	1513	+	CACGTG
	PIF1	1508	1513	-	CACGTG
	PIF1	1520	1525	+	CACGTG
	PIF1	1520	1525	-	CACGTG
XP_010102578.1	PIF1	1495	1500	+	CACGTG
	PIF1	1495	1500	-	CACGTG

	BEE2	1495	1500	+	CACGTG
	BEE2	1495	1500	-	CACGTG
	Dof2	120	124	-	AAAGC
XP_010107463.1	Dof3	1309	1314	+	AAAGCG
	ARR11	1722	1729	+	AAGATACG
	ATHB-16	898	905	+	TAATAATT
	ABF3	479	485	-	ACGTGTC
	Dof2	643	647	-	AAAGC
XP_024030012.1	ATHB-16	1806	1813	+	TAATAATT
	Dof2	753	757	-	AAAGC
	Dof2	847	851	-	AAAGC
	CDF2	1317	1323	-	AAAAAGT
	CDF2	1737	1743	+	AAAAAGT
XP_010094609.1	Dof3	1949	1954	+	AAAGCG
	ATHB-16	1513	1520	-	TAATAATT
	Dof2	1235	1239	-	AAAGC
	Dof2	1949	1953	+	AAAGC
	Dof2	1981	1985	+	AAAGC
XP_024023501.1	Dof3	1464	1469	-	AAAGCG
	Dof2	11	15	-	AAAGC
	Dof2	115	119	-	AAAGC
	Dof2	133	137	+	AAAGC
	Dof2	330	334	-	AAAGC
XP_010112647.2	ATHB-7	432	442	-	TCAATGATTGA
	Dof2	63	67	+	AAAGC

	Dof2	211	215	+	AAAGC
	Dof2	404	408	+	AAAGC
	Dof2	519	523	+	AAAGC
XP_024023006.1	ATHB-16	415	422	-	TAATAATT
	PIF1	1234	1239	+	CACGTG
	PIF1	1234	1239	-	CACGTG
	BEE2	1234	1239	+	CACGTG
	BEE2	1234	1239	-	CACGTG
XP_024022961.1	ATHB-16	889	896	+	TAATAATT
	CDF2	1282	1291	-	AAAAAAGTGA
	ABF3	1570	1576	+	ACGTGTC
	ABF3	1658	1664	+	ACGTGTC
	Dof2	352	356	-	AAAGC
XP_010087133.1	Dof3	875	880	-	AAAGCG
	Dof3	1237	1242	+	AAAGCG
	ATHB-16	316	323	-	TAATAATT
	ATHB-16	823	830	+	TAATAATT
	ATHB-16	1438	1445	+	TAATAATT
XP_024023462.1	Dof2	438	442	+	AAAGC
	Dof2	469	473	+	AAAGC
	CDF2	1899	1905	-	AAAAAGT
	MaB1A	438	442	+	AAAGC
	MaB1A	469	473	+	AAAGC
XP_024023382.1	Dof2	230	234	+	AAAGC
	Dof2	1444	1448	-	AAAGC

	Dof2	1465	1469	-	AAAGC
	CDF2	1577	1583	+	AAAAAGT
	MaB1A	230	234	+	AAAGC
XP_024025182.1	Dof3	1658	1663	+	AAAGCG
	Dof2	17	21	+	AAAGC
	Dof2	598	602	-	AAAGC
	Dof2	1269	1273	+	AAAGC
	Dof2	1347	1351	-	AAAGC
XP_010087454.1	Dof3	553	558	-	AAAGCG
	ATHB-16	491	498	+	TAATAATT
	Dof2	151	155	+	AAAGC
	Dof2	169	173	-	AAAGC
	Dof2	224	228	-	AAAGC
XP_010108436.1	Dof2	1000	1004	-	AAAGC
	Dof2	1349	1353	+	AAAGC
	Dof2	1926	1930	+	AAAGC
	CDF2	521	527	-	AAAAAGT
	CDF2	543	549	-	AAAAAGT
XP_010094405.1	Dof3	301	306	-	AAAGCG
	Dof2	302	306	-	AAAGC
	Dof2	529	533	+	AAAGC
	Dof2	1873	1877	-	AAAGC
	MaB1A	302	306	-	AAAGC
XP_010101004.1	ATHB-16	1614	1621	-	TAATAATT
	Dof2	267	271	-	AAAGC

	Dof2	413	417	-	AAAGC
	Dof2	450	454	-	AAAGC
	Dof2	482	486	-	AAAGC
XP_010104824.1	Dof3	440	445	-	AAAGCG
	ATHB-16	973	980	+	TAATAATT
	ATHB-16	1387	1394	+	TAATAATT
	ATHB-16	1848	1855	+	TAATAATT
	Dof2	285	289	-	AAAGC
XP_010109635.1	Dof3	749	754	+	AAAGCG
	Dof2	31	35	-	AAAGC
	Dof2	749	753	+	AAAGC
	Dof2	1155	1159	-	AAAGC
	CDF2	1603	1609	-	AAAAAGT
XP_024020016.1	ATHB-16	747	754	-	TAATAATT
	ATHB-16	1227	1234	+	TAATAATT
	Dof2	347	351	+	AAAGC
	Dof2	614	618	-	AAAGC
	Dof2	883	887	-	AAAGC
XP_010088252.1	Dof3	1726	1731	-	AAAGCG
	Dof3	1951	1956	-	AAAGCG
	ATHB-16	165	172	+	TAATAATT
	ATHB-16	194	201	-	TAATAATT
	ATHB-16	1553	1560	-	TAATAATT
XP_010102581.1	Dof2	32	36	+	AAAGC
	Dof2	950	954	+	AAAGC

	Dof2	1406	1410	-	AAAGC
	Dof2	1641	1645	-	AAAGC
	Dof2	1681	1685	-	AAAGC
XP_024017058.1	ABF3	815	821	+	ACGTGTC
	Dof2	643	647	-	AAAGC
	CDF2	1004	1010	-	AAAAAGT
	MaB1A	643	647	-	AAAGC
	PBF	643	647	-	AAAGC
XP_024032474.1	PIF1	234	239	+	CACGTG
	PIF1	234	239	-	CACGTG
	BEE2	234	239	+	CACGTG
	BEE2	234	239	-	CACGTG
	ABF3	235	241	+	ACGTGTC
XP_010102577.1	Dof2	9	13	-	AAAGC
	Dof2	426	430	+	AAAGC
	Dof2	1705	1709	-	AAAGC
	CDF2	224	230	-	AAAAAGT
	CDF2	817	823	+	AAAAAGT
XP_010104406.1	ATHB-16	800	807	-	TAATAATT
	Dof2	96	100	-	AAAGC
	Dof2	314	318	-	AAAGC
	Dof2	621	625	-	AAAGC
	Dof2	689	693	+	AAAGC
XP_024030227.1	Dof3	1691	1696	+	AAAGCG
	ATHB-16	1949	1956	-	TAATAATT

	Dof2	267	271	-	AAAGC
	Dof2	875	879	+	AAAGC
	Dof2	920	924	+	AAAGC
XP_024026054.1	ATHB-16	20	27	-	TAATAATT
	ATHB-16	72	79	-	TAATAATT
	Dof2	890	894	+	AAAGC
	Dof2	1040	1044	+	AAAGC
	Dof2	1433	1437	+	AAAGC
XP_024020043.1	Dof2	858	862	-	AAAGC
	Dof2	1361	1365	-	AAAGC
	MaB1A	858	862	-	AAAGC
	MaB1A	1361	1365	-	AAAGC
	PBF	858	862	-	AAAGC
XP_010102366.1	Dof3	1100	1105	+	AAAGCG
	ATHB-16	534	541	-	TAATAATT
	PIF1	707	712	+	CACGTG
	PIF1	707	712	-	CACGTG
	BEE2	707	712	+	CACGTG
XP_024024700.1	Dof2	63	67	-	AAAGC
	Dof2	748	752	-	AAAGC
	Dof2	1443	1447	+	AAAGC
	CDF2	282	288	+	AAAAAGT
	MaB1A	63	67	-	AAAGC
XP_024019354.1	Dof3	465	470	+	AAAGCG
	Dof3	718	723	+	AAAGCG

	Dof3	1750	1755	-	AAAGCG
	Dof3	1833	1838	+	AAAGCG
	Dof2	99	103	-	AAAGC
XP_010094388.1	NAC058	447	454	-	ACACGCAA
	ATHB-16	69	76	+	TAATAATT
	ABF3	523	529	+	ACGTGTC
	Dof2	41	45	-	AAAGC
	Dof2	411	415	+	AAAGC
XP_010108435.1	ATHB-16	1973	1980	+	TAATAATT
	ATHB-16	1977	1984	-	TAATAATT
	PIF1	1012	1017	+	CACGTG
	PIF1	1012	1017	-	CACGTG
	PIF1	1313	1318	+	CACGTG
XP_024032012.1	Dof3	259	264	-	AAAGCG
	ATHB-16	515	522	-	TAATAATT
	PIF1	304	309	+	CACGTG
	PIF1	304	309	-	CACGTG
	BEE2	304	309	+	CACGTG
XP_010101001.1	Dof3	1049	1054	+	AAAGCG
	Dof3	1079	1084	+	AAAGCG
	ATHB-16	919	926	-	TAATAATT
	PIF1	1468	1473	+	CACGTG
	PIF1	1468	1473	-	CACGTG
XP_024031300.1	PIF1	23	28	+	CACGTG
	PIF1	23	28	-	CACGTG

	MYC4	22	28	-	CACGTGC
	BEE2	23	28	+	CACGTG
	BEE2	23	28	-	CACGTG
XP_010102583.1	Dof2	743	747	+	AAAGC
	Dof2	993	997	+	AAAGC
	Dof2	1052	1056	+	AAAGC
	Dof2	1305	1309	+	AAAGC
	CDF2	489	495	-	AAAGC
XP_010089434.1	Dof2	147	151	+	AAAGC
	Dof2	1055	1059	+	AAAGC
	Dof2	1383	1387	+	AAAGC
	Dof2	1393	1397	+	AAAGC
	CDF2	983	989	-	AAAAAGT
XP_010104408.1	Dof3	1175	1180	+	AAAGCG
	Dof3	1757	1762	-	AAAGCG
	Dof2	3	7	+	AAAGC
	Dof2	75	79	-	AAAGC
	Dof2	986	990	+	AAAGC
XP_024026540.1	Dof3	1422	1427	-	AAAGCG
	Dof2	67	71	-	AAAGC
	Dof2	1173	1177	-	AAAGC
	Dof2	1405	1409	-	AAAGC
	Dof2	1423	1427	-	AAAGC
XP_024022630.1	Dof2	402	406	+	AAAGC
	Dof2	405	409	-	AAAGC

	HAT5	1953	1960	-	CAATTATT
	MNB1A	402	406	+	AAAGC
	MNB1A	405	409	-	AAAGC

Supplementary Table S4. Log_2-transformed expression values of 50 *Morus notabilis PLA* genes across five tissues (bud, leaf, staminate flower, pistillate flower, root) and under drought and salt stress treatments, grouped by subfamily (*pPLA*, *PLA2*, *PLA1*). Tissue expression values are $2^{-\Delta Ct}$ (log_2-transformed); stress values are $2^{-\Delta\Delta Ct}$ (log_2-transformed). Data are organized by subfamily: *pPLA* (22 genes), *PLA2* (9 genes), *PLA1* (19 genes). These values were used to generate the heatmaps in Figure 5.

ID	bud	leaf	staminate flower	pistillate flower	root	Drought/Control	Salinity/Control	Group-I
XP_010101003.1	-0.84	-1.19	-2.83	-2.61	-0.02	-1.60	0.87	pPLA
XP_010101001.1	3.81	-1.79	-0.64	2.12	6.06	6.93	-3.30	pPLA
XP_010101004.1	-6.21	-0.15	-7.63	-6.88	-2.34	-2.52	-1.91	pPLA
XP_024022961.1	-2.05	6.10	-4.01	-3.02	-0.08	-0.68	2.72	pPLA
XP_024020016.1	-1.86	-3.87	4.70	-0.53	-2.74	-0.98	5.43	pPLA
XP_010089434.1	-2.03	-3.40	-0.22	-1.62	3.37	0.36	-0.17	pPLA
XP_024026054.1	-8.32	-3.44	-6.41	-3.34	-2.26	-0.06	-7.96	pPLA
XP_010094388.1	8.52	-9.39	11.80	8.82	3.19	-3.66	-1.51	pPLA
XP_010109903.1	-2.74	-0.02	-4.76	-6.21	-7.08	-0.36	5.21	pPLA
XP_010102581.1	1.73	1.02	0.55	1.12	5.80	0.57	4.66	pPLA
XP_010102583.1	-3.00	-5.71	-1.44	-3.82	2.75	-0.43	-1.47	pPLA
XP_024025182.1	-7.14	-7.26	-7.17	-5.79	-6.03	-3.53	-1.93	pPLA
XP_010112647.2	-7.34	-2.48	-8.17	-6.83	-5.76	0.36	-7.24	pPLA
XP_024032012.1	0.35	-4.50	-2.57	-1.90	-1.59	1.69	-2.37	pPLA
XP_010094405.1	2.68	3.25	-3.21	-4.46	0.54	-2.41	1.52	pPLA

XP_024020043.1	-7.19	-6.87	-4.99	-8.28	-3.84	-1.95	0.11	pPLA
XP_010090405.1	7.08	-4.97	6.35	5.00	7.73	2.68	2.70	pPLA
XP_024023462.1	-1.11	-3.32	-0.48	-0.71	4.91	1.34	2.30	pPLA
XP_010109635.1	-6.18	-6.49	-5.49	-7.72	-5.17	3.44	-3.38	pPLA
XP_024032474.1	-0.14	1.74	2.89	1.93	4.59	-3.02	-3.24	pPLA
XP_024024700.1	-4.50	-6.92	-1.24	-2.07	-3.25	-6.85	1.11	pPLA
XP_024023501.1	-4.18	-5.77	4.18	-2.55	2.69	-7.52	0.03	pPLA
ID	bud	leaf	male	female	root	Drought/Control	Salinity/Control	Group-II
XP_010112575.1	-1.92	-4.99	3.25	-2.33	6.26	-2.51	-8.64	PLA2
XP_024030012.1	6.11	-6.44	5.92	3.79	5.40	-3.95	-1.97	PLA2
XP_024023382.1	-3.00	-4.72	-8.41	-0.13	7.13	3.74	-5.50	PLA2
XP_010094609.1	-3.55	-2.94	2.47	-2.55	3.12	2.62	4.61	PLA2
XP_024019354.1	-4.38	-8.92	-6.90	-3.37	-6.99	5.41	6.62	PLA2
XP_024023006.1	-0.83	-2.17	3.63	6.54	9.65	0.32	-5.32	PLA2
XP_024030227.1	1.92	1.12	2.87	5.41	3.58	0.31	-3.44	PLA2
XP_010087454.1	0.39	0.29	-4.04	0.36	2.55	-0.03	-6.78	PLA2
XP_010088252.1	4.08	-1.13	3.54	1.80	-0.01	-2.28	-2.72	PLA2
ID	bud	leaf	SF	PF	root	Drought/Control	Salinity/Control	Group-III
XP_010102578.1	0.52	-15.49	-5.31	-9.46	-0.47	-0.06	-0.19	PLA1
XP_010102577.1	12.15	4.36	10.18	8.23	5.22	-4.96	1.72	PLA1
XP_024031300.1	5.48	-0.12	-5.23	2.27	1.22	-1.69	0.95	PLA1
XP_024022632.1	10.90	-0.35	3.87	5.76	6.54	-7.85	-6.23	PLA1
XP_024022630.1	3.09	3.32	3.93	2.34	2.58	-3.31	-0.21	PLA1
XP_010104276.2	1.85	-6.83	1.13	-0.89	1.55	-2.24	0.93	PLA1
XP_010104824.1	4.10	0.10	3.65	0.70	-0.04	-2.59	-0.49	PLA1

XP_010105205.2	-4.79	-6.63	-7.17	-4.93	-3.21	0.57	-7.65	PLA1
XP_010104409.1	2.55	-2.40	4.95	4.49	-2.53	-6.34	-0.95	PLA1
XP_010107463.1	-5.02	-6.29	-2.85	-4.82	-5.01	-6.67	-16.26	PLA1
XP_010104406.1	3.43	-0.54	4.77	3.83	3.77	-1.06	0.03	PLA1
XP_010104408.1	2.37	3.78	3.55	2.81	2.64	-3.80	-2.34	PLA1
XP_024026540.1	2.32	3.71	3.61	3.11	2.58	4.40	-1.37	PLA1
XP_010108436.1	-3.11	-2.39	-2.65	8.92	0.87	3.14	-5.00	PLA1
XP_010108435.1	-6.06	1.80	-11.11	-7.97	-7.69	-0.11	4.45	PLA1
XP_024025676.1	2.74	-0.95	2.00	1.66	5.43	0.62	-4.54	PLA1
XP_024017058.1	2.22	-3.91	1.86	0.90	8.22	-1.77	-0.89	PLA1
XP_010087133.1	4.93	-6.03	0.91	-0.02	2.35	-6.63	-1.11	PLA1
XP_010102366.1	4.54	-3.41	3.50	2.24	5.26	0.29	-2.07	PLA1
